# Supplementary material for: Gut bacteria-derived sphingolipids alter innate immune responses to oral cholera vaccine antigens
Source: Nat Commun. 2025 Dec 12;17:638. doi: 10.1038/s41467-025-67388-y (PMC12816596; doi:10.1038/s41467-025-67388-y)
Supplement: Supplementary file 1 — Supplementary Information [file 41467_2025_67388_MOESM1_ESM.pdf]

**TITLE:** Gut bacteria-derived sphingolipids alter innate immune responses to oral cholera vaccine antigens

**Table of Contents**

**Supplementary Figure 1.** Microbial diversity by age and sex.....2

**Supplementary Figure 2.** Microbial diversity by memory B cell response. .....3

**Supplementary Figure 3.** Rarefaction curve of microbiome data. .....4

**Supplementary Figure 4.** Number of bacterial genomes mapped to significant CAGs. .....5

**Supplementary Figure 5.** Top strains associated with CT-specific MBC responses after OCV. .....6

**Supplementary Figure 6.** Phylogenetic tree of *Bacteroides xylanisolvens* strains.....8

**Supplementary Figure 7.** Ceramide levels in *B. xylanisolvens* cultures with and without myriocin.....**9**

**Supplementary Figure 8.** THP-1 derived macrophages exposed to SL-reduced lysate induces IL-6 response.....10

**Supplementary Figure 9.** Lipid treatment of THP-1 derived macrophages did not alter cell viability. .....11

**Supplementary Figure 10.** Reducing phospholipids in lipid extracts increases cytokine responses to heat-killed JBK70. .....12

**Supplementary Figure 11.** *B. koreensis* SL-containing pretreatment on THP-1 macrophages increases cytokine responses to heat-killed JBK70. .....13

**Supplementary Figure 12.** Preconditioning with Bx SL-containing lipid treatment triggers innate immune pathway activation. .....14

**Supplementary Figure 13.** Differential gene expression in macrophages after preconditioning with Bx SL-containing or SL-reduced lipid fractions and stimulation with JBK70.....15

**Supplementary Figure 14.** Stimulated PBMCs have increased inflammatory cytokine responses to JBK70 following SL-containing lipid pretreatment. .....16

**Supplementary Table 1.** Number of Significant CAGS per vaccine response.....17

**Supplementary Table 2.** Summary of strains associated with specific vaccine response measures. .....18

**Supplementary Table 3.** Top lipid species found in human stool, comparison between vaccine responders and non-responders.....19

**Supplementary Table 4.** Ceramides found in *B. xylanisolvens* grown in BHI-supplemented media with or without spt inhibitor myriocin. .....20

**Supplementary Table 5.** Differential gene expression attributed to treatment with heat-killed *V. cholerae* vaccine strain, JBK70. .....21

**Supplementary Table 6.** Top blastp result for *Bacteroides fragilis* serine palmitoyltransferase gene. .....22

**STORMS Checklist**.....23

**Study Protocol**.....36

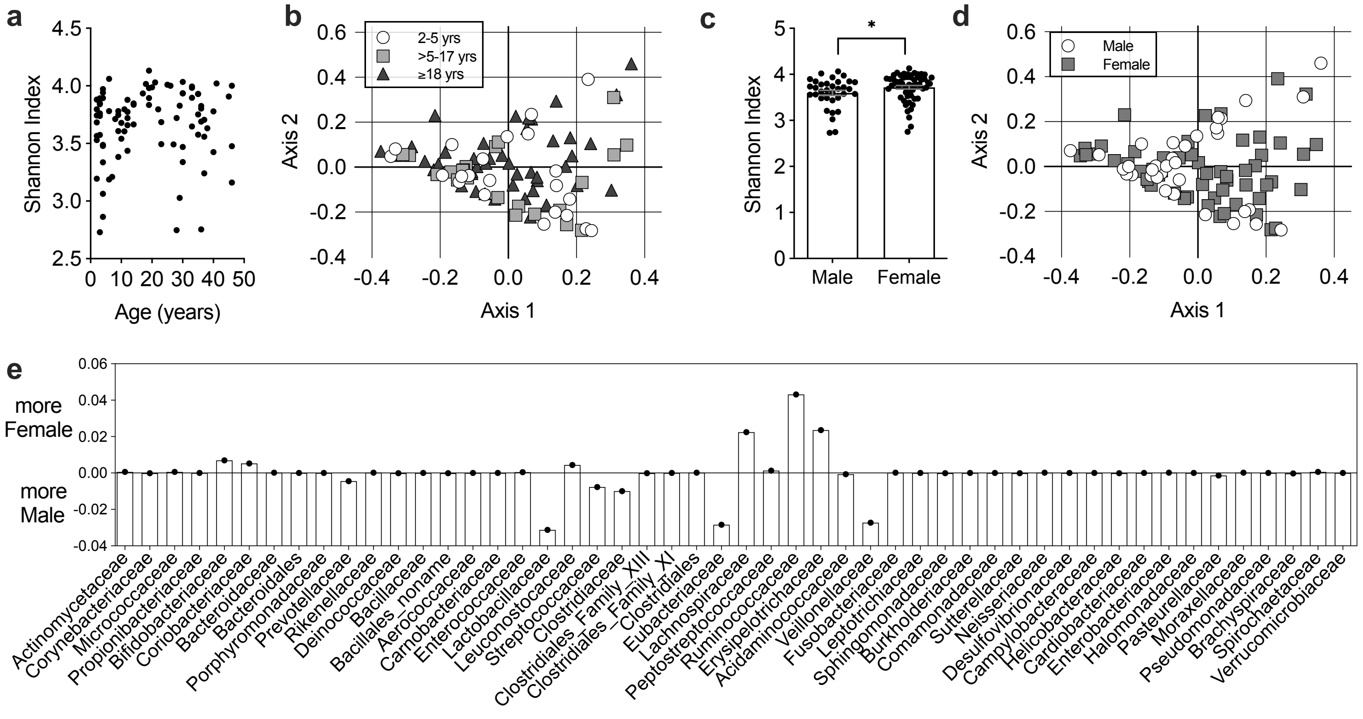


**Supplementary Figure 1.** Microbial diversity by age and sex.
(**a**) Shannon Index by age. No difference was observed between participants by Kruskal-Wallis testing when samples were divided by age group (2-5, >5-17, ≥18 years of age, P>0.05). (**b**) Beta diversity between age groups measured by Bray-Curtis Dissimilarity, Principal Component Analysis (PCA). No difference was observed in statistical testing between age groups via Kruskal-Wallis testing (P>0.05). (**c**) Shannon diversity according to participant sex. Bars represent mean with SD, and male and female participants were found to have a significantly different Shannon Index by Mann-Whitney U testing (*, P=0.03). (**d**) Beta diversity between sexes measured by Bray-Curtis Dissimilarity. No difference was found between sexes by Mann-Whitney U testing (P>0.05). Dots in each graph represent one participant. (**e**) Abundance differences in baseline gut microbiota populations by sex (family level shown). Positive values on the X-axis represent a greater proportion of gut microbes in that family in female participants. All families with abundance <1% in this study population are shown.

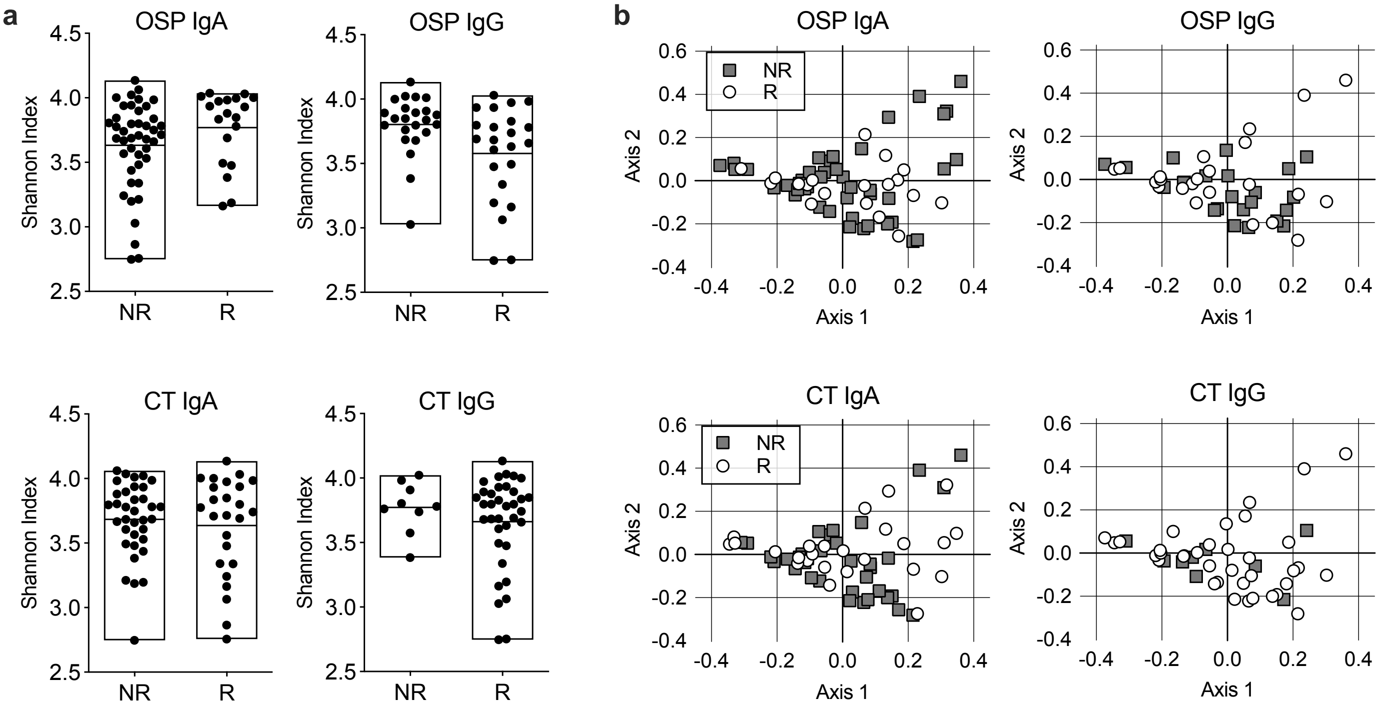


**<u>Supplementary Figure 2.</u>** <u>Microbial diversity by memory B cell response</u>.
(**a**) Shannon index of vaccine responders (R) and nonresponders (NR) by MBC-specific responses. Box represents max and min with middle line at the mean. No difference was found between groups via Mann-Whitney U testing (P>0.05 in each group). (**b**) PCA demonstrating beta diversity between R and NR measured by Bray-Curtis Dissimilarity within each vaccine response measure. No difference was seen between R and NR groups by Mann-Whitney U testing (P>0.05). Dots in each graph represent one participant.

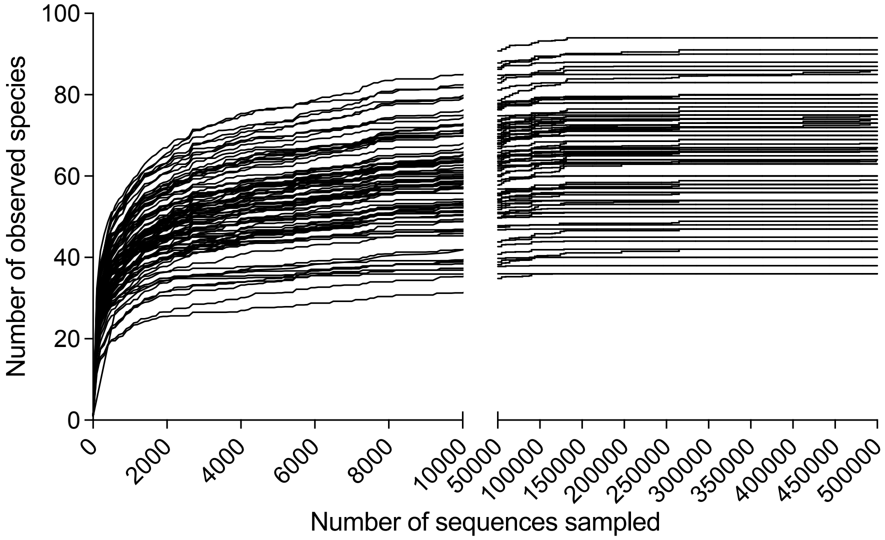


**Supplementary Figure 3.** Rarefaction curve of microbiome data.
N=89 fecal samples were analyzed for microbiome. Each line represents an individual sample. Rarefaction performed on species-level identification of microbiome species.

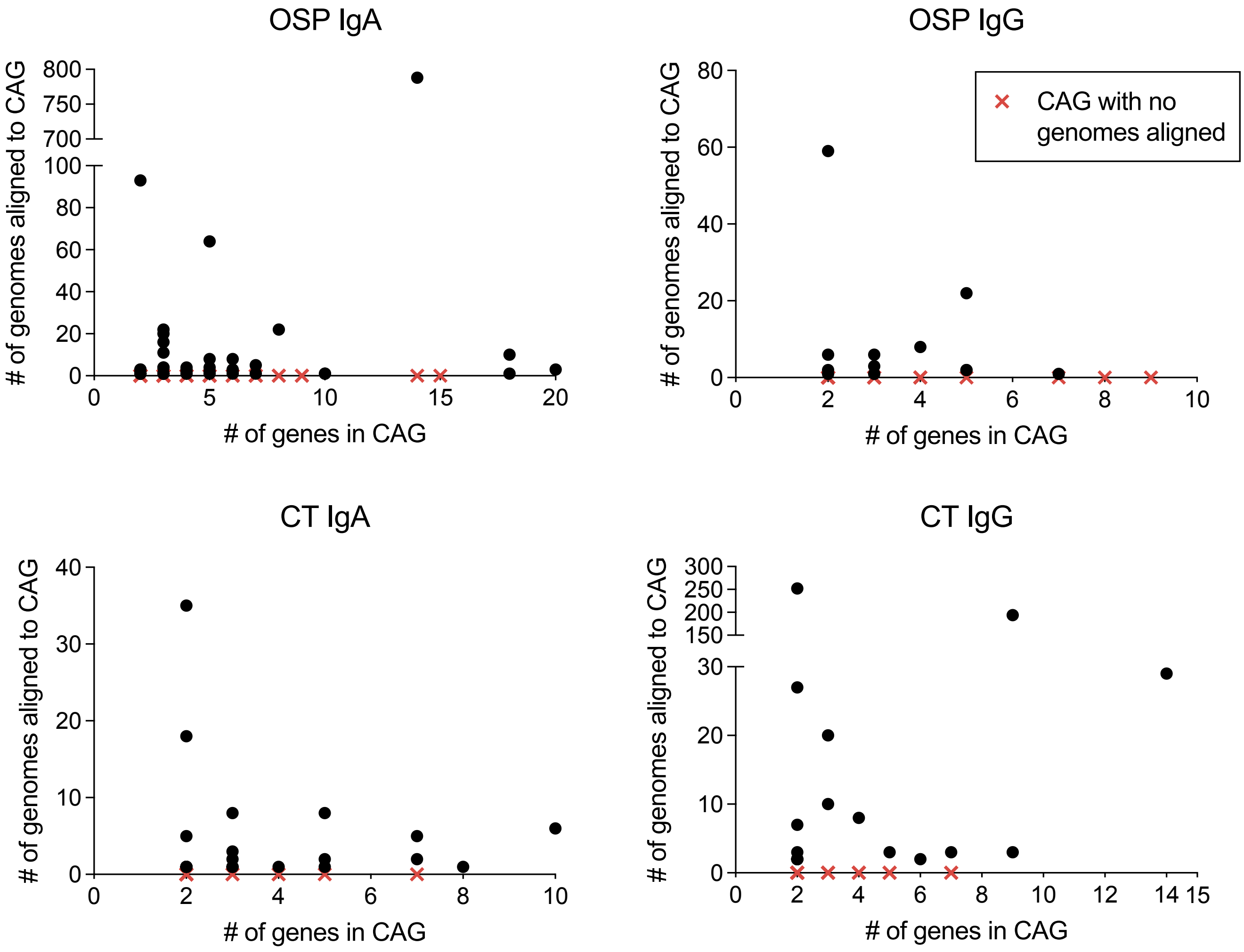


**<u>Supplementary Figure 4.</u>** <u>Number of bacterial genomes mapped to significant CAGs.</u>
NCBI reference bacterial genomes were mapped to CAGs that were found to be associated with memory B cell responses. Only significant CAGs associated with vaccine response are shown, and each is represented as a circle or X. Significant CAGs are defined as a CAG containing ≥2 genes with a q value of ≤0.1 for an association with the listed vaccine response measure. Red x's indicate CAGs that had no genomes aligned. The x-axis indicates the number of genes within each CAG while the y-axis represents the number of reference genomes to which genes in those CAGs align to with >90% sequence identity.

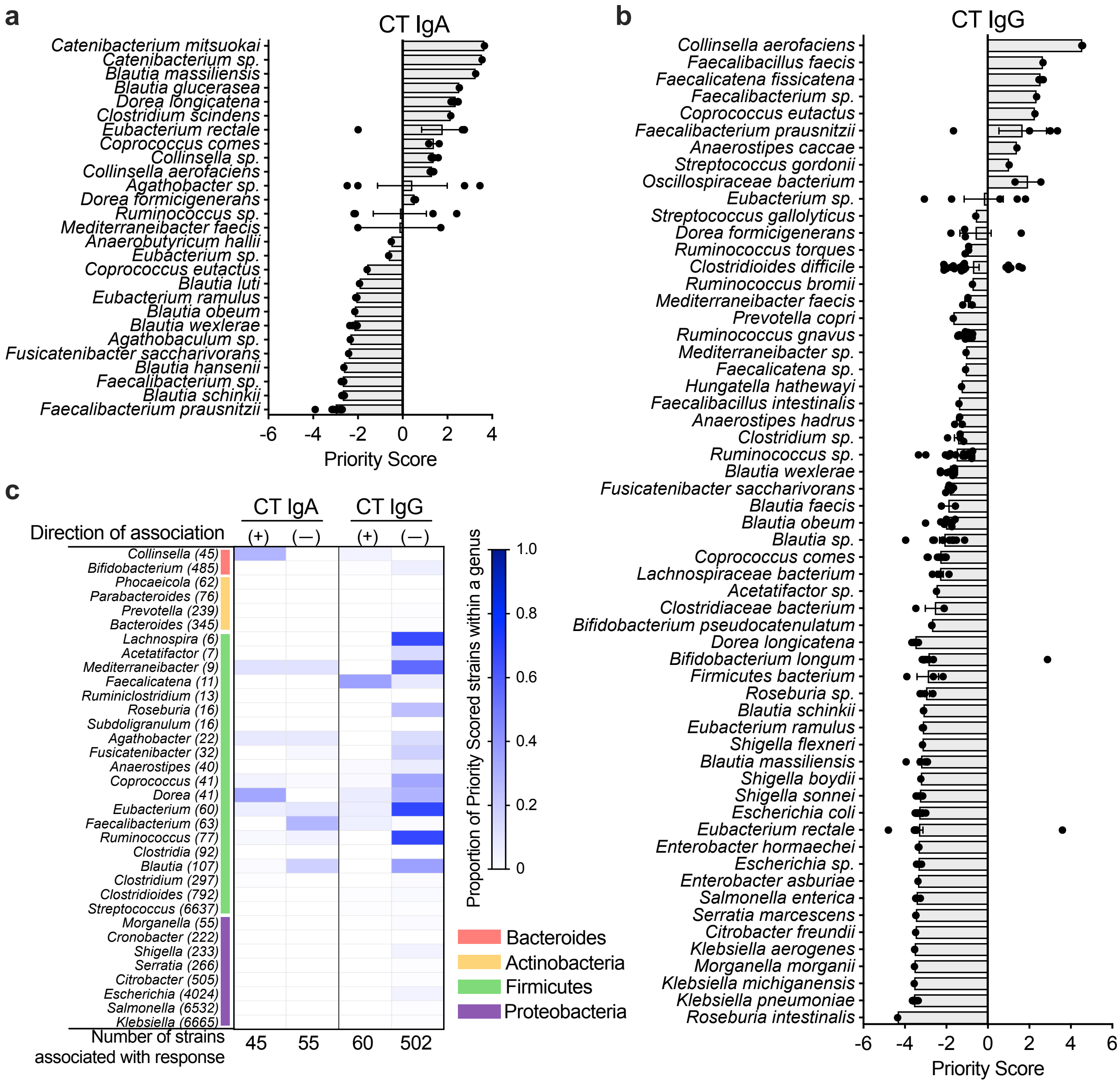


**Supplementary Figure 5.** Top strains associated with CT-specific MBC responses after OCV. Species containing strains with a priority score greater than 0.5 are shown for (**a**) CT IgA and (**b**) CT IgG MBC responses. Each dot indicates the priority score of a specific strain within the species listed on the left in each row. For each species, bars represent mean with SEM of priority scored strains. Full strain data and priority scores for each MBC response are shown in **Supplementary Data 4-5**. (**c**) Proportion of strains found in our study population within each genus that were associated with a specific MBC response with positive (+) and (-) associations. Genera that contained 5 or more strains with significant CAGs were plotted with shading representing the percentage of total strains that contained significant CAGs. Parentheses indicate the total number of distinct strains in each genus found in our study population. Phylum identifications are shown in the colored vertical bar.

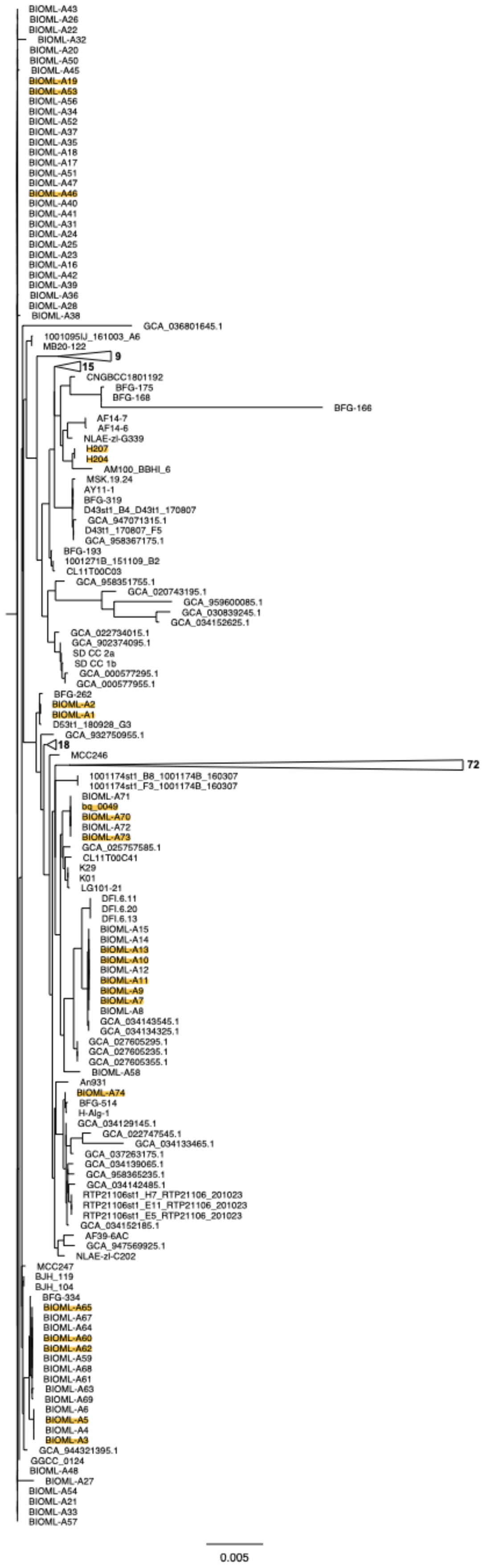

BIOML-A43
BIOML-A26
BIOML-A22
BIOML-A32
BIOML-A20
BIOML-A50
BIOML-A45
BIOML-A19
BIOML-A53
BIOML-A56
BIOML-A34
BIOML-A52
BIOML-A37
BIOML-A35
BIOML-A18
BIOML-A17
BIOML-A51
BIOML-A47
BIOML-A46
BIOML-A40
BIOML-A41
BIOML-A31
BIOML-A24
BIOML-A25
BIOML-A23
BIOML-A16
BIOML-A42
BIOML-A39
BIOML-A36
BIOML-A28
BIOML-A38
GCA_036801645.1
1001095IJ_161003_A6
MB20-122
9
15
CNGBCC1801192
BFG-175
BFG-168
BFG-166
AF14-7
AF14-6
NLAE-zl-G339
H207
H204
AM100_BBHI_6
MSK.19.24
AY11-1
BFG-319
D43st1_B4_D43t1_170807
GCA_947071315.1
D43t1_170807_F5
GCA_958367175.1
BFG-193
1001271B_151109_B2
CL11T00C03
GCA_958351755.1
GCA_020743195.1
GCA_959600085.1
GCA_030839245.1
GCA_034152625.1
GCA_022734015.1
GCA_902374095.1
SD CC 2a
SD CC 1b
GCA_000577295.1
GCA_000577955.1
BFG-262
BIOML-A2
BIOML-A1
D53t1_180928_G3
GCA_932750955.1
18
MCC246
72
1001174st1_B8_1001174B_160307
1001174st1_F3_1001174B_160307
BIOML-A71
bq_0049
BIOML-A70
BIOML-A72
BIOML-A73
GCA_025757585.1
CL11T00C41
K29
K01
LG101-21
DFI.6.11
DFI.6.20
DFI.6.13
BIOML-A15
BIOML-A14
BIOML-A13
BIOML-A10
BIOML-A12
BIOML-A11
BIOML-A9
BIOML-A7
BIOML-A8
GCA_034143545.1
GCA_034134325.1
GCA_027605295.1
GCA_027605235.1
GCA_027605355.1
BIOML-A58
An931
BIOML-A74
BFG-514
H-Alg-1
GCA_034129145.1
GCA_022747545.1
GCA_034133465.1
GCA_037263175.1
GCA_034139065.1
GCA_958365235.1
GCA_034142485.1
RTP21106st1_H7_RTP21106_201023
RTP21106st1_E11_RTP21106_201023
RTP21106st1_E5_RTP21106_201023
GCA_034152185.1
AF39-6AC
GCA_947569925.1
NLAE-zl-C202
MCC247
BJH_119
BJH_104
BFG-334
BIOML-A65
BIOML-A67
BIOML-A64
BIOML-A60
BIOML-A62
BIOML-A59
BIOML-A68
BIOML-A61
BIOML-A63
BIOML-A69
BIOML-A6
BIOML-A5
BIOML-A4
BIOML-A3
GCA_944321395.1
GGCC_0124
BIOML-A48
BIOML-A27
BIOML-A54
BIOML-A21
BIOML-A33
BIOML-A57
0.005

**Supplementary Figure 6.** Phylogenetic tree of *Bacteroides xylanisolvens* strains.
*Bacteroides xylanisolvens* NCBI genomes are shown, and *B. xylanisolvens* strains from the stool of our study population are highlighted. Tree assembly was performed using GToTree and drawn with FigTree.

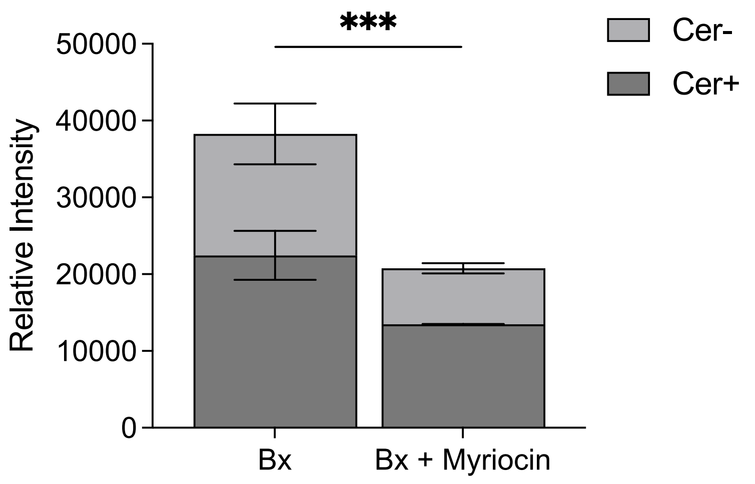


**Supplementary Figure 7.** Ceramide levels in *B. xylanisolvens* cultures with and without myriocin.
*B. xylanisolvens* (Bx) was grown in BHI supplemented media in anaerobic conditions with or without the *spt* inhibitor, myriocin. N=3 technical replicates per condition. Lipids were then extracted using the Bligh-Dyer and measured using hydrophilic interaction liquid chromatography and ion mobility-mass spectrometry. Data is shown as the total intensity of detected positive (Cer+) and negative (Cer-) mode ceramides tested in triplicate. Intensity refers to the signal strength of an ion detected by the mass spectrometer and is proportional to the abundance of that ion. Ions are measured in either negative or positive mode based on their chemical structure and functional groups. Two-tailed unpaired t test with SEM is shown. ***, P=0.0007.

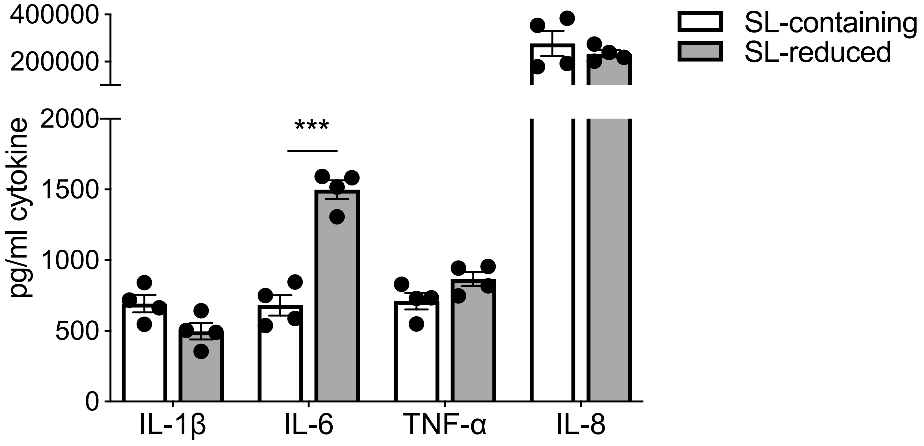


**Supplementary Figure 8.** THP-1 derived macrophages exposed to SL-reduced lysate induces IL-6 response. Cytokine responses were measured in supernatant of THP-1 derived macrophages incubated with *Bacteroides xylanisolvens* lysate that was grown with or without myriocin, SL-reduced and SL-containing, respectively, for 18 hours. Statistical analysis was performed using multiple two-tailed unpaired t tests with FDR two-stage set-up method of Benjamini, Kriege and Yekutieli. ***, P=0.000154, q= 0.000467. Bars indicate mean with SEM.

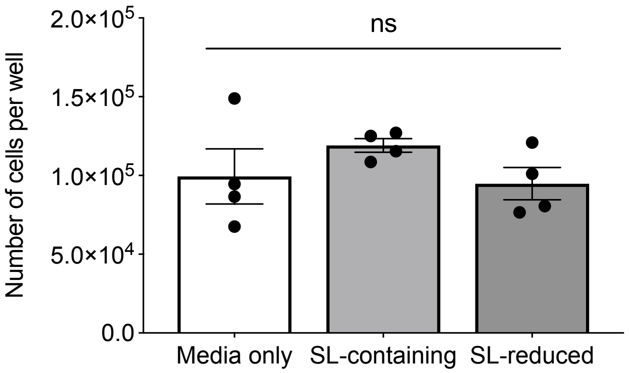


**Supplementary Figure 9.** Lipid treatment of THP-1 derived macrophages did not alter cell viability.
The number of adherent cells were counted after THP-1 derived macrophages were incubated with *B. xylanisolvens* lipid extracts that was grown with or without myriocin, SL-reduced and SL-containing, respectively, for 24 hours. Statistical analysis was performed using one-way ANOVA. ns, $P>0.05$. Bars indicate mean with SEM.

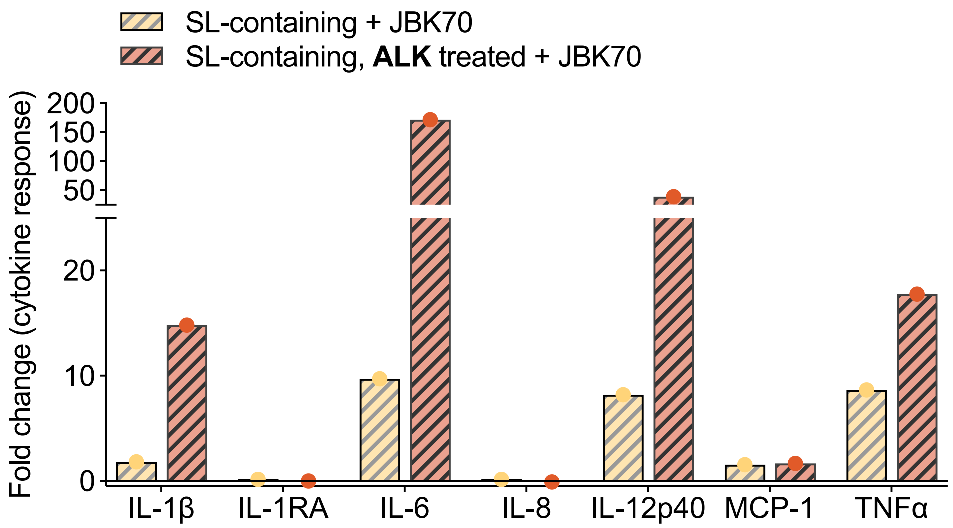


**Supplementary Figure 10.** Reducing phospholipids in lipid extracts increases cytokine responses to heat-killed JBK70.

THP-1 derived macrophage fold change cytokine response to heat killed JBK70 after preconditioning with *B. xylanisolvens* lipids with or without a mild alkaline hydrolysis treatment (ALK, a treatment that reduces non-sphingolipid phospholipids) demonstrated increases in response compared to SL-containing fractions alone. Cytokines were measured using a 15-cytokine multiplex assay.

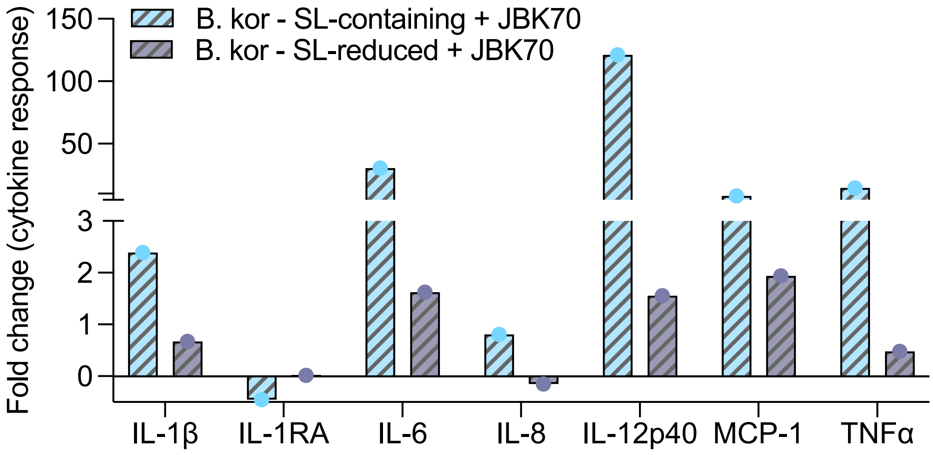


<u>**Supplementary Figure 11.** *B. koreensis* SL-containing pretreatment on THP-1 macrophages increases cytokine responses to heat-killed JBK70.</u>

Fold change in cytokine response of THP-1 derived macrophages to heat-killed JBK70 after preconditioning with SL-containing and SL-reduced *Bacteroides koreensis* (B. kor) lipids. *B. koreensis* lipids were Preconditioning was conducted for 24 hours and stimulation for 18 hours, with methods the same as in Bx experiments with identical conditions. Cytokines were measured using a 15-cytokine multiplex assay.

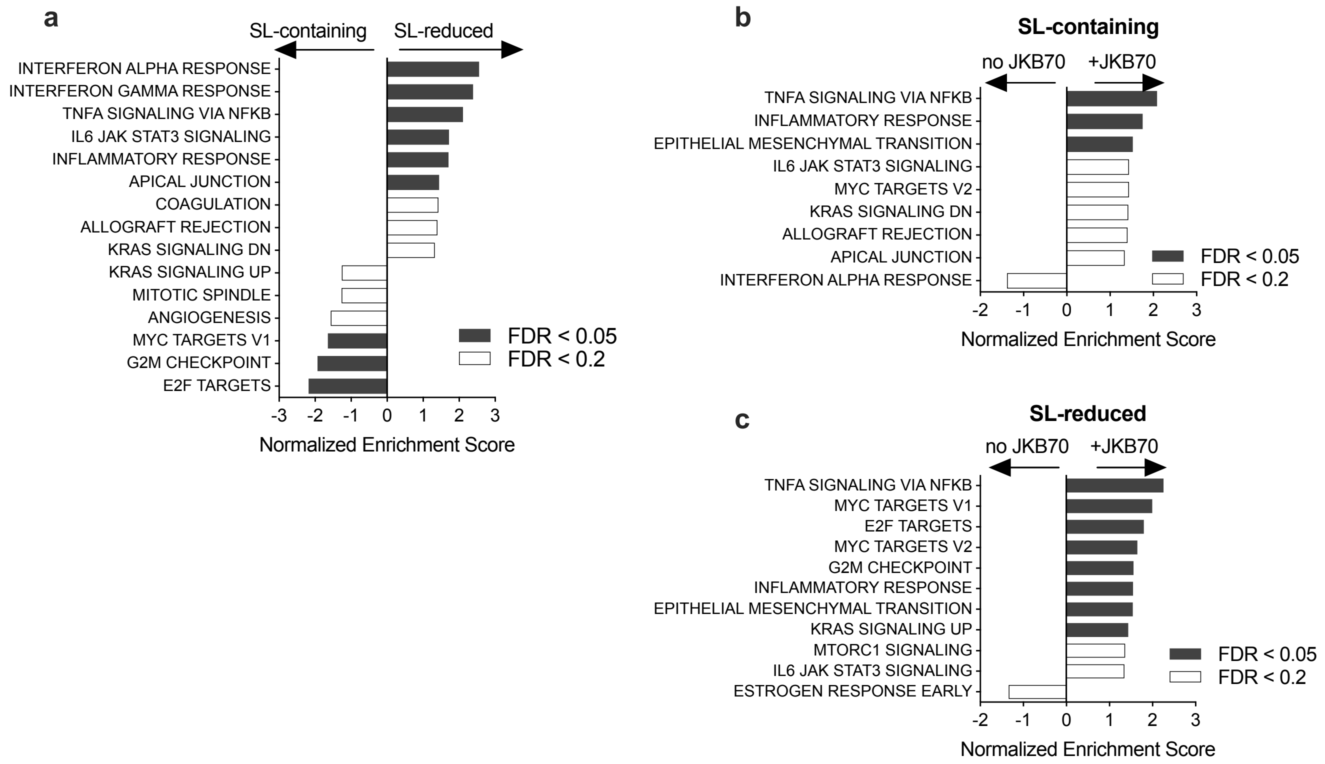


**Supplementary Figure 12.** Preconditioning with Bx SL-containing lipid treatment triggers innate immune pathway activation.

Pathway analysis and gene categories generated using the gene set enrichment analysis method on fold change values of all genes present in the dataset is shown. Pathways enriched between (**a**) SL-containing and SL-reduced preconditioning without JBK70 stimulation, (**b**) SL-containing fraction with and without JBK70 stimulation, (**c**) and SL-reduced with and without JBK70 stimulation.

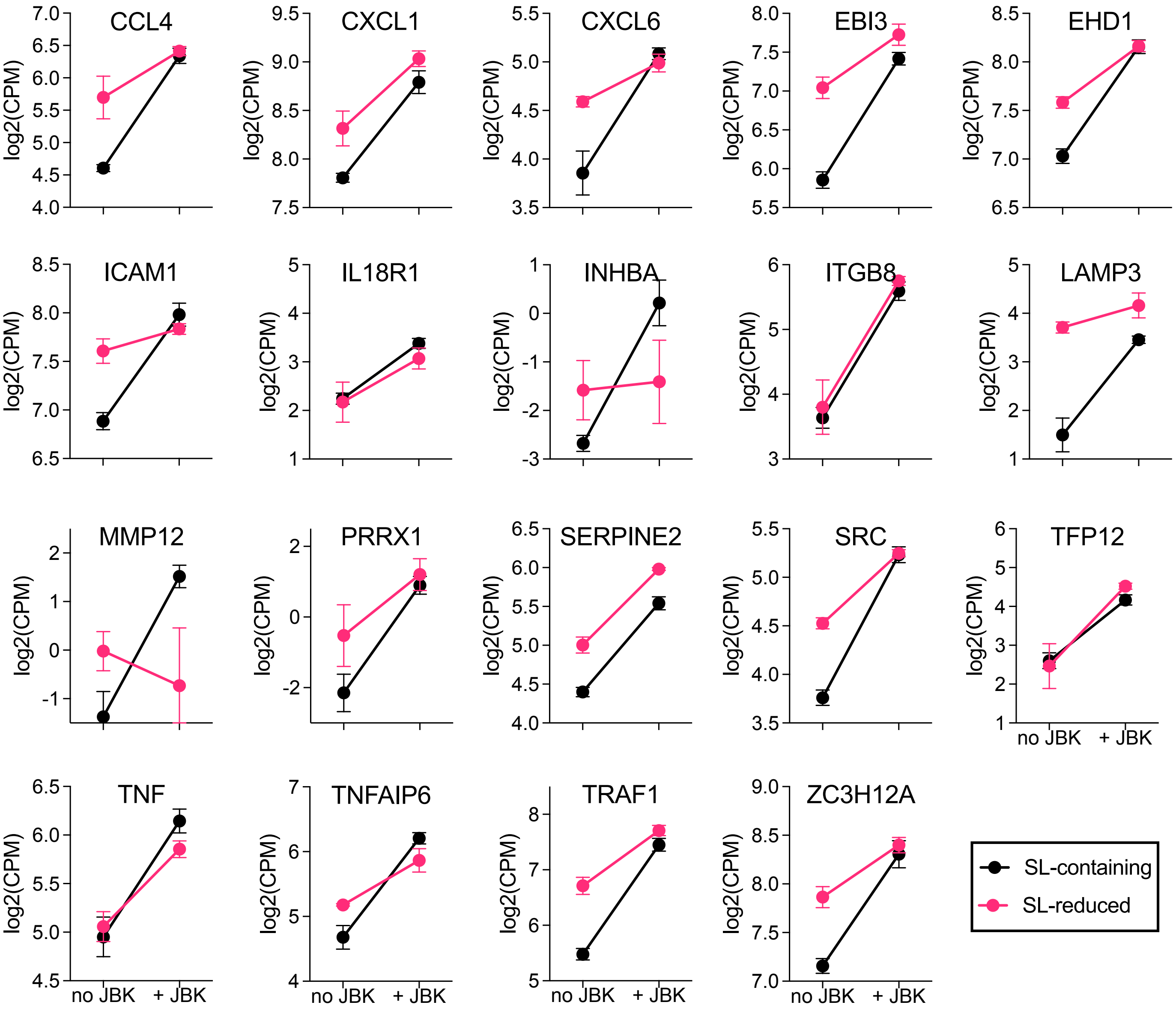


**Supplementary Figure 13.** Differential gene expression in macrophages after preconditioning with Bx SL-containing or SL-reduced lipid fractions and stimulation with JBK70.
Gene expression is shown as log2 counts per million (CPM) of the 19 genes in the enriched pathways identified using gene set enrichment analysis, described in Figure 5C-D.

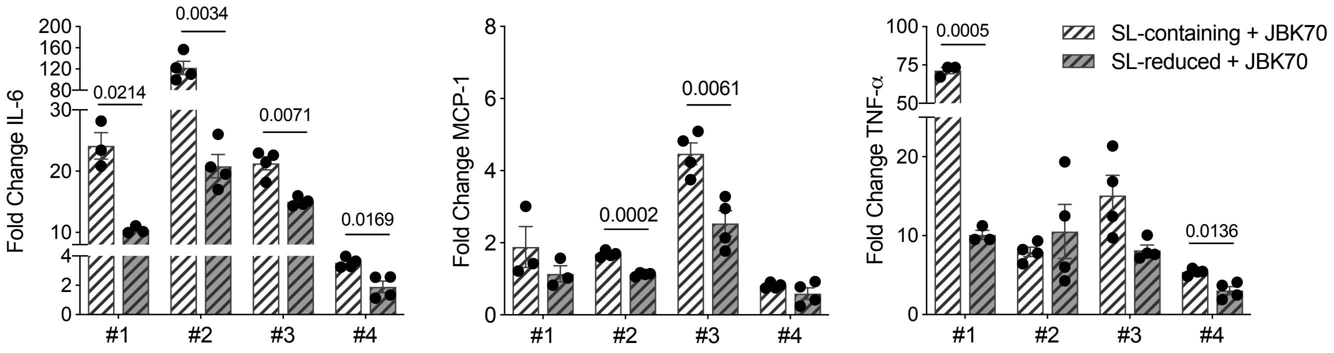


**<u>Supplementary Figure 14.</u>** <u>Stimulated PBMCs have increased inflammatory cytokine responses to JBK70 following SL-containing lipid pretreatment.</u>
Fold change in cytokine response in supernatant from peripheral blood mononuclear cells (PBMCs) after preconditioning with *B. xylanisolvens* lipid extracts and treated with heat-killed JBK70. Bars represent mean with SEM. Two-tailed unpaired t-test comparing fold change. Each number represents a different PBMC donor. #1 = 31 yr female; #2 = 25 yr male; #3 = 36 yr female; and #4 = 35 yr male. Three to four technical replicates were performed on each experimental condition depending on the PBMCs available after thawing.

**<u>Supplementary Table 1.</u>** <u>Number of Significant CAGS per vaccine response.</u>
CAGs with ≥ 2 genes and an association with each MBC, FDR q-value ≤ 0.1.

| | Number significant CAGs total *(positive/negative association)* | Number CAGs with no genes aligning to reference strains | est coeff mean - pos (std dev) | est coeff mean - neg (std dev) | wald mean - pos (std dev) | wald mean - neg (std dev) |
|---|---|---|---|---|---|---|
| OSP IgA | 169 *(166/3)* | 123 (73%) | 0.915 (0.284) | -1.03 (0.390) | 2.82 (0.456) | -2.54 (0.0719) |
| OSP IgG | 48 (40/8) | 36 (75%) | 0.975 (-0.351) | -1.06 (0.613) | 2.68 (0.253) | -2.49 (0.183) |
| CT IgA | 52 (40/12) | 28 (54%) | 0.942 (0.256) | -0.890 (0.327) | 2.79 (0.4423) | -2.53 (0.260) |
| CT IgG | 54 (41/13) | 32 (59%) | 0.871 (-0.343) | -0.93 (-0.381) | 2.71 (0.356) | -2.51 (0.201) |

**Supplementary Table 2.** Summary of strains associated with specific vaccine response measures.
Counts of strains positively and negatively associated with each vaccine response measure based on alignment of the 323 significant CAGs. Mean priority scores for positively and negatively associated strains, along with standard deviations (SD), are also shown by vaccine response measure.

| | Number of positively associated strains | Number of negatively associated strains | Mean positive priority score (SD) | Mean negative priority score (SD) |
|---|---|---|---|---|
| OSP IgA | 1107 | 0 | 3.90 (1.37) | NA |
| OSP IgG | 112 | 1 | 1.39 (0.33) | -0.704 (NA) |
| CT IgA | 45 | 55 | 1.81 (0.95) | -2.30 (0.70) |
| CT IgG | 59 | 503 | 1.20 (1.17) | -2.78 (0.95) |

**Supplementary Table 3.** Top lipid species found in human stool, comparison between vaccine responders and non-responders.
N=9 responders and n=7 nonresponder fecal samples were analyzed for targeted lipidomics. The top lipid species with P-values < 0.05 are shown (two-tailed multiple Mann-Whitney tests performed in Prism). Highlighted in gray are sphingolipid derivatives. CE: cholesterol Ester; CER: ceramides; DAG: Diacylglycerol; DCER: dihydroceramides; FFA: Free fatty acids; HCER: Hexosylceramides; LCER: Lactosylceramides; SM: Sphingomyelin; TAG: Triacylglycerol.

| Lipid species | P value | Responder average in nmol/g | Nonresponder average in nmol/g | R / NR | Log2(FC) |
|---|---|---|---|---|---|
| DAG(14:1/18:1) | 0.000699 | 2.788455556 | 0.566257143 | 4.924363 | 2.299937 |
| CE(20:2) | 0.001486 | 1.075155556 | 0.046757143 | 22.99447 | 4.523215 |
| FFA(22:5) | 0.005245 | 21.71195556 | 8.194085714 | 2.649711 | 1.405835 |
| SM(24:0) | 0.005245 | 15.13698889 | 8.441571429 | 1.793148 | 0.842495 |
| DAG(16:0/20:4) | 0.007692 | 0.062133333 | 0.530271429 | 0.117173 | -3.093292 |
| CER(18:0) | 0.007867 | 9.917266667 | 2.114242857 | 4.690694 | 2.229801 |
| FFA(14:1) | 0.007867 | 5.917133333 | 2.596457143 | 2.278926 | 1.188354 |
| FFA(20:5) | 0.007867 | 23.01973333 | 6.206414286 | 3.709023 | 1.891039 |
| FFA(20:3) | 0.007867 | 20.05677778 | 8.258542857 | 2.42861 | 1.280131 |
| CER(16:0) | 0.007867 | 38.96262222 | 11.10477143 | 3.508638 | 1.810911 |
| TAG52:6-FA18:3 | 0.007955 | 0.0326 | 2.321357143 | 0.014044 | -6.153953 |
| TAG54:8-FA18:3 | 0.007955 | 0.072811111 | 6.6817 | 0.010897 | -6.519913 |
| TAG56:8-FA20:4 | 0.008479 | 0.065911111 | 0.2152 | 0.306278 | -1.707084 |
| CER(24:0) | 0.011538 | 8.478433333 | 2.172142857 | 3.903258 | 1.964679 |
| CE(18:0) | 0.011538 | 15.25155556 | 5.268357143 | 2.894936 | 1.533531 |
| FFA(20:4) | 0.011538 | 22.73613333 | 8.182042857 | 2.778784 | 1.474454 |
| HCER(22:1) | 0.016084 | 3.413288889 | 0.955071429 | 3.573857 | 1.837482 |
| DAG(14:1/16:0) | 0.016434 | 0.872722222 | 0.330857143 | 2.637761 | 1.399314 |
| CER(18:1) | 0.016434 | 4.435866667 | 0.927157143 | 4.784374 | 2.25833 |
| CE(24:0) | 0.016434 | 2.940388889 | 1.062585714 | 2.767202 | 1.468428 |
| CE(20:4) | 0.016434 | 4.887988889 | 1.5717 | 3.110001 | 1.636915 |
| CER(24:1) | 0.016434 | 12.52642222 | 3.108071429 | 4.030288 | 2.010883 |
| FFA(20:2) | 0.016434 | 20.1705 | 6.257657143 | 3.223331 | 1.688552 |
| SM(16:0) | 0.016434 | 29.08417778 | 6.628857143 | 4.38751 | 2.133402 |
| FFA(18:1) | 0.016434 | 698.3271333 | 261.0786857 | 2.674777 | 1.419418 |
| TAG50:2-FA16:1 | 0.017395 | 0.228033333 | 0.772685714 | 0.295118 | -1.760637 |
| TAG44:2-FA18:2 | 0.022378 | 0.222 | 0.616542857 | 0.360072 | -1.473642 |
| CE(16:1) | 0.022902 | 2.544922222 | 1.0517 | 2.419818 | 1.274898 |
| SM(18:0) | 0.022902 | 7.657077778 | 1.151542857 | 6.649408 | 2.733226 |
| CER(22:0) | 0.022902 | 7.772133333 | 1.781485714 | 4.362726 | 2.12523 |
| FFA(16:1) | 0.022902 | 37.2408 | 15.97442857 | 2.331276 | 1.22112 |
| CE(20:5) | 0.028934 | 1.666933333 | 0.236585714 | 7.04579 | 2.816762 |
| TAG52:6-FA18:2 | 0.031031 | 0.023411111 | 0.589485714 | 0.039714 | -4.654192 |
| FFA(18:4) | 0.031119 | 6.032 | 2.147571429 | 2.808754 | 1.48993 |
| FFA(22:2) | 0.031119 | 16.55538889 | 5.197928571 | 3.184997 | 1.671292 |
| FFA(22:4) | 0.031119 | 12.24203333 | 5.312614286 | 2.304333 | 1.204349 |
| CE(16:0) | 0.031119 | 26.69787778 | 12.64071429 | 2.112055 | 1.078647 |
| TAG40:0-FA14:0 | 0.033042 | 12.46605556 | 35.62857143 | 0.349889 | -1.51503 |
| CE(22:4) | 0.041259 | 0.683766667 | 0.086971429 | 7.861969 | 2.974891 |
| TAG51:1-FA17:0 | 0.041259 | 0.343255556 | 0.377542857 | 0.909183 | -0.137357 |
| CER(26:0) | 0.041783 | 0.834288889 | 0.501057143 | 1.665057 | 0.735572 |
| CER(20:0) | 0.041783 | 1.941033333 | 0.5628 | 3.448887 | 1.786131 |
| CE(18:1) | 0.041783 | 36.26437778 | 15.82571429 | 2.291484 | 1.196282 |
| TAG40:0-FA12:0 | 0.045105 | 14.38835556 | 30.2747 | 0.47526 | -1.073211 |
| DCER(20:1) | 0.048077 | 0.048366667 | 0.461871429 | 0.104719 | -3.255406 |

**Supplementary Table 4.** Ceramides found in *B. xylanisolvens* grown in BHI-supplemented media with or without spt inhibitor myriocin.
Data is lipids measured using Bligh-Dyer lipid extraction and liquid chromatography-mass spectrometry, using technical triplicates of *Bacteroides xylanisolvens* cultures.

| | Bx, no myriocin (SL-containing) mean±SD | Bx, with myriocin (SL-reduced) mean±SD | Two-tailed Unpaired T test P-value |
|---|---|---|---|
| Cer(d18:1/18:1)_[M+H]+ | 2737 (±907.6) | 1292 (±208.8) | 0.0548 |
| Cer(d18:1/16:0)_[M-H]- | 959.4 (±563.7) | 144.8 (±23.93) | 0.0667 |

**Supplementary Table 5.** Differential gene expression attributed to treatment with heat-killed *V. cholerae* vaccine strain, JBK70.
Overlapping 10 genes that were differentially expressed with heat-killed *V. cholerae* JBK70 in both SL-containing and SL-reduced pretreatments. N=3 technical replicates were performed on THP1 macrophages. FDR-adjusted values are shown, according to linear modeling using kmFIT.

| gene | symbol | SL-containing fraction, FDR value | SL-containing fraction, log fold change | SL-reduced fraction, FDR value | SL-reduced fraction, log fold change |
|---|---|---|---|---|---|
| ENSG00000125538 | IL1B | 0.17151695 | 2.35526258 | 0.12437583 | 1.50700287 |
| ENSG00000169429 | CXCL8 | 0.17151695 | 1.78529097 | 0.1642097 | 1.30157571 |
| ENSG00000183160 | TMEM119 | 0.17151695 | 1.10296962 | 0.12437583 | 1.00653128 |
| ENSG00000184557 | SOCS3 | 0.17151695 | 1.73025723 | 0.12696118 | 1.1609136 |
| ENSG00000253831 | ETV3L | 0.17151695 | 1.2774653 | 0.12696118 | 1.25354486 |
| ENSG00000104267 | CA2 | 0.18017546 | -1.7457118 | 0.16603796 | -1.4411888 |
| ENSG00000213949 | ITGA1 | 0.18533197 | 1.00840318 | 0.16603796 | 1.10402886 |
| ENSG00000131459 | GFPT2 | 0.21265351 | 2.78391162 | 0.16299971 | 1.71056026 |
| ENSG00000161896 | IP6K3 | 0.23015157 | 4.10878118 | 0.22460769 | 2.63048695 |
| ENSG00000121807 | CCR2 | 0.24722885 | -1.8325467 | 0.13895514 | -1.1325317 |

**Supplementary Table 6.** Top blastp result for *Bacteroides fragilis* serine palmitoyltransferase gene.
Blastp results for *Bacteroides fragilis* spt (encoded by GenBank# EXZ60402.1) in the *B. xylanisolven* strains found in study metagenomics. Highlighted are the strains with score (bits) > 500 and E-values < E-50.

| **Reference** | **Strain** | **Blastp result** | **Blastp result - description** | **Score (bits)** | **E-value** |
|---|---|---|---|---|---|
| GCA_006546965.1 | H207 | QDH57762.1 | aminotransferase class I/II-fold pyridoxal phosphate-d... | 840 | 0 |
| GCA_008710235.1 | H204 | KAA9036003.1 | aminotransferase class I/II-fold pyridoxal phosphate... | 840 | 0 |
| GCA_009102805.1 | BIOML-A5 | KAB6411518.1 | aminotransferase class I/II-fold pyridoxal phosphate... | 842 | 0 |
| GCA_009102665.1 | BIOML-A13 | KAB6369277.1 | aminotransferase class I/II-fold pyridoxal phosphate... | 294 | 2E-97 |
| GCA_009102705.1 | BIOML-A11 | KAB6401651.1 | aminotransferase class I/II-fold pyridoxal phosphate... | 294 | 2E-97 |
| GCA_009102755.1 | BIOML-A7 | KAB6420522.1 | aminotransferase class I/II-fold pyridoxal phosphate... | 294 | 2E-97 |
| GCA_009102675.1 | BIOML-A10 | KAB6389565.1 | aminotransferase class I/II-fold pyridoxal phosphate... | 294 | 2E-97 |
| GCA_009093775.1 | BIOML-A70 | KAB6092873.1 | aminotransferase class I/II-fold pyridoxal phosphate... | 295 | 5E-98 |
| GCA_004167295.1 | bq_0049 | RYT14463.1 | aminotransferase class I/II-fold pyridoxal phosphate-d... | 295 | 5E-98 |
| GCA_009102845.1 | BIOML-A3 | KAB6430851.1 | aminotransferase class I/II-fold pyridoxal phosphate... | 842 | 0 |
| GCA_009102725.1 | BIOML-A9 | KAB6394303.1 | aminotransferase class I/II-fold pyridoxal phosphate... | 294 | 2E-97 |
| GCA_009102025.1 | BIOML-A60 | KAB6129010.1 | aminotransferase class I/II-fold pyridoxal phosphate... | 840 | 0 |
| GCA_009095545.1 | BIOML-A65 | KAB6103963.1 | aminotransferase class I/II-fold pyridoxal phosphate... | 840 | 0 |
| GCA_009102045.1 | BIOML-A62 | KAB6132819.1 | aminotransferase class I/II-fold pyridoxal phosphate... | 840 | 0 |
| GCA_009101535.1 | BIOML-A2 | KAB6434403.1 | aminotransferase class I/II-fold pyridoxal phosphate... | 295 | 5E-98 |
| GCA_009101515.1 | BIOML-A19 | KAB6331313.1 | glycine C-acetyltransferase [Bacteroides xylanisolvens] | 219 | 2E-68 |
| GCA_009101785.1 | BIOML-A1 | KAB6451913.1 | aminotransferase class I/II-fold pyridoxal phosphate... | 295 | 5E-98 |
| GCA_009093695.1 | BIOML-A74 | KAB6081657.1 | aminotransferase class I/II-fold pyridoxal phosphate... | 295 | 6E-98 |
| GCA_009153275.1 | BIOML-A73 | KAB6084048.1 | aminotransferase class I/II-fold pyridoxal phosphate... | 295 | 5E-98 |
| GCA_009102225.1 | BIOML-A46 | KAB6208771.1 | aminotransferase class I/II-fold pyridoxal phosphate... | 295 | 5E-98 |
| GCA_009101555.1 | BIOML-A53 | KAB6188534.1 | aminotransferase class I/II-fold pyridoxal phosphate... | 295 | 5E-98 |

| STORMS Checklist for Microbiome Research | | | | | | |
|---|---|---|---|---|---|---|
| Version: | 1.03 | | | | | |
| **Number** | **Item** | **Recommendation** | **Item Source** | **Additional Guidance** | **Yes/No/NA** | **Comments or location in manuscript** |
| **Abstract** | | | | | | |
| 1.0 | Structured or Unstructured Abstract | Abstract should include information on background, methods, results, and conclusions in structured or unstructured format. | STORMS | | Yes | |
| 1.1 | Study Design | State study design in abstract. | STORMS | See 3.0 for additional information on study design. | Yes | |
| 1.2 | Sequencing methods | State the strategy used for metagenomic classification. | STORMS | For example, targeted 16S by qPCR or sequencing, shotgun metagenomics, metatranscriptomics, etc. | Yes | |
| 1.3 | Specimens | Describe body site(s) studied. | STORMS | | Yes | |
| **Introduction** | | | | | | |
| 2.0 | Background and Rationale | Summarize the underlying background, scientific evidence, or theory driving the current hypothesis as well as the study objectives. | STORMS | | Yes | |
| 2.1 | Hypotheses | State the pre-specified hypothesis. If the study is exploratory, state any pre-specified study objectives. | STORMS | | Yes | The objective is stated in the introduction |
| **Methods** | | | | | | |
| 3.0 | Study Design | Describe the study design. | STORMS | Observational (Case-Control, Cohort, Cross-sectional survey, etc.) or Experimental (Randomized controlled trial, Non-randomized controlled trial, etc.). For a brief description of common study designs see: DOI: 10.11613/BM.2014.022<br><br>If applicable, describe any blinding (e.g. single or double-blinding) used in the course of the study. | Yes | Study design and protocol are provided. This is also previously described in parent study (Dash et al., 2024 – msphere. doi: 10.1128/msphere.00565-23) |

| | | | | | | |
|---|---|---|---|---|---|---|
| 3.1 | Participants | State what the population of interest is, and the method by which participants are sampled from that population. Include relevant information on physiological state of the subjects or stage in the life history of disease under study when participants were sampled. | STORMS | Examples of the population of interest could be: adults with no chronic health conditions, adults with type II diabetes, newborns, etc. This is the total population to whom the study is hoped to be generalizable to. The sampling method describes how potential participants were selected from that population.<br><br>If the participants are from a substudy of a larger study, provide a brief description of that study and cite that study.<br><br>Clearly state how cases and controls are defined.<br><br>An example of relevant physiological state might be pre/post menopausal for a vaginal microbiome study; examples of stage in the life history of disease could be whether specimens were collected during active or dormant disease, or before or after treatment. | Yes | Study design and protocol are provided. This is also previously described in parent study (Dash et al., 2024 – msphere. doi: 10.1128/msphere.00565-23) |
| 3.2 | Geographic location | State the geographic region(s) where participants were sampled from. | MIxS: geographic location (country and/or sea,region) | Geographic coordinates can be reported to prevent potential ambiguities if necessary. | Yes | Study design and protocol are provided. This is also previously described in parent study (Dash et al., 2024 – msphere. doi: 10.1128/msphere.00565-23) |
| 3.3 | Relevant Dates | State the start and end dates for recruitment, follow-up, and data collection. | STORMS | Recruitment is the period in which participants are recruited for the study. In longitudinal studies, follow-up is the date range in which participants are asked to complete a specific assessment. Finally, data collection is the total period in which data is being collected from participants including during initial recruitment through all follow-ups. | Yes | Study design and protocol are provided. This is also previously described in parent study (Dash et al., 2024 – msphere. doi: 10.1128/msphere.00565-23) |

| | | | | | | |
|---|---|---|---|---|---|---|
| 3.4 | Eligibility criteria | List any criteria for inclusion and exclusion of recruited participants. | Modified STROBE | Among potential recruited participants, how were some chosen and others not? This could include criteria such as sex, diet, age, health status, or BMI.<br><br>If there is a primary and validation sample, describe inclusion/exclusion criteria for each. | Yes | Study design and protocol are provided. This is also previously described in parent study (Dash et al., 2024 – msphere. doi: 10.1128/msphere.00565-23) |
| 3.5 | Antibiotics Usage | List what is known about antibiotics usage before or during sample collection. | STORMS | If participants were excluded due to current or recent antibiotics usage, state this here.<br><br>Other factors (e.g. proton pump inhibitors, probiotics, etc.) that may influence the microbiome should also be described as well. | Yes | Study design and protocol are provided. This is also previously described in parent study (Dash et al., 2024 – msphere. doi: 10.1128/msphere.00565-23) |
| 3.6 | Analytic sample size | Explain how the final analytic sample size was calculated, including the number of cases and controls if relevant, and reasons for dropout at each stage of the study. This should include the number of individuals in whom microbiome sequencing was attempted and the number in whom microbiome sequencing was successful. | STORMS | Consider use of a flow diagram (see template at https://stormsmicrobiome.org/figures). Also state sample size in abstract.<br><br>If power analysis was used to calculate sample size, describe those calculations. | NA | Sample size was designed for original study period; previously described (Dash et al., 2024 – msphere. doi: 10.1128/msphere.00565-23) |
| 3.7 | Longitudinal Studies | For longitudinal studies, state how many follow-ups were conducted, describe sample size at follow-up by group or condition, and discuss any loss to follow-up. | STORMS | If there is loss to follow-up, discuss the likelihood that drop-out is associated with exposures, treatments, or outcomes of interest. | NA | Follow-up studies were conducted in the original study. |
| 3.8 | Matching | For matched studies, give matching criteria. | Modified STROBE | "Matched" refers to matching between comparable study participants as cases and controls or exposed / unexposed.<br><br>Indicate whether participants were individual or frequency matched and in what ratio were they matched (e.g. 1 case to 1 control). | NA | |
| 3.9 | Ethics | State the name of the institutional review board that approved the study and protocols, protocol number and date of approval, and procedures for obtaining informed consent from participants. | STORMS | | Yes | This is provided in the methods and also previously described in parent study (Dash et al., 2024 – msphere. doi: 10.1128/msphere.00565-23) |

| | | | | | | |
|---|---|---|---|---|---|---|
| 4.0 | Laboratory methods | State the laboratory/center where laboratory work was done. | STORMS | Provide a reference to complete lab protocols if previously published elsewhere such as on protocols.io. Note any modifications of lab protocols and the reason for protocol modifications. | Yes | |
| 4.1 | Specimen collection | State the body site(s) sampled from and how specimens were collected. | MIxS: sample collection device or method; host body site | Use terms from the Uber-anatomy Ontology (https://www.ebi.ac.uk/ols/ontologies/uberon) to describe body sites in a standardized format. | Yes | |
| 4.2 | Shipping | Describe how samples were stored and shipped to the laboratory. | STORMS | Include length of time from collection to receipt by the lab and if temperature control was used during shipping. | Yes | |
| 4.3 | Storage | Describe how the laboratory stored samples, including time between collection and storage and any preservation buffers or refrigeration used. | STORMS | State where each procedure or lot of samples was done if not all in the same place.<br><br>Include reagent/lot/catalogue #s for storage buffers. | Yes | This is provided in the methods section. |
| 4.4 | DNA extraction | Provide DNA extraction method, including kit and version if relevant. | MIxS: nucleic acid extraction | If any DNA quantification methods were used prior to DNA amplification or at the pooling step of library preparation, state so here. | Yes | This is provided in the methods section. |
| 4.5 | Human DNA sequence depletion or microbial DNA enrichment | Describe whether human DNA sequence depletion or enrichment of microbial or viral DNA was performed. | STORMS | | NA | Not used |
| 4.6 | Primer selection | Provide primer selection and DNA amplification methods as well as variable region sequenced (if applicable). | MIxS: pcr primers | | NA | Not used |
| 4.7 | Positive Controls | Describe any positive controls (mock communities) if used. | STORMS | If used, should be deposited under guidance provided in the 8.X items. | NA | Not used |
| 4.8 | Negative Controls | Describe any negative controls if used. | STORMS | If used, should be deposited under guidance provided in the 8.X items. | NA | Not used |
| 4.9 | Contaminant mitigation and identification | Provide any laboratory or computational methods used to control for or identify microbiome contamination from the environment, reagents, or laboratory. | STORMS | Includes filtering of reagents and other steps to minimize contamination. It is relevant to state whether the specimens of interest have low microbial load, which makes contamination especially relevant. | NA | Samples were processed using aseptic techniques and sterile consumables. No computational methods were used to mitigate contamination. Samples do not have low microbial load |

| | | | | | | |
|---|---|---|---|---|---|---|
| 4.10 | Replication | Describe any biological or technical replicates included in the sequencing, including which steps were replicated between them. | STORMS | Replication may be biological (redundant biological specimens) or technical (aliquots taken at different stages of analysis) and used in extraction, sequencing, preprocessing, and/or data analysis. | Yes | All fecal samples are independent biolgocail samples. Technical replicates were not performed on sequencing samples. |
| 4.11 | Sequencing strategy | Major divisions of strategy, such as shotgun or amplicon sequencing. | MIxS: sequencing method | For amplicon sequencing (for example, 16S variable region), state the region selected. State the model of sequencer used. | NA | Not used |
| 4.12 | Sequencing methods | State whether experimental quantification was used (QMP/cell count based, spike-in based) or whether relative abundance methods were applied. | STORMS | These include read length, sequencing depth per sample (average and minimum), whether reads are paired, and other parameters. | Yes | This is provided in the methods section. |
| 4.13 | Batch effects | Detail any blocking or randomization used in study design to avoid confounding of batches with exposures or outcomes. Discuss any likely sources of batch effects, if known. | STORMS | Sources of batch effects include sample collection, storage, library preparation, and sequencing and are commonly unavoidable in all but the smallest of studies. | Yes | Microbiome samples were uniformily stored, processed, extracted, and sequenced in one batch. |
| 4.14 | Metatranscriptomics | Detail whether any mRNA enrichment was performed and whether/how retrotranscription was performed prior to sequencing. Provide size range of isolated transcripts. Describe whether the sequencing library was stranded or not. Provide details on sequencing methods and platforms. | STORMS | Provide details on any internal standards which may have been used as well as parameters and versions of any software or databases used. | Yes | This is provided in the methods section. |
| 4.15 | Metaproteomics | Detail which protease was used for digestion. Provide details on proteomic methods and platforms (e.g. LC-MS/MS, instrument type, column type, mass range, resolution, scan speed, maximum injection time, isolation window, normalised collision energy, and resolution). | STORMS | Provide details on any internal standards which may have been used as well as parameters and versions of any software or databases used. | NA | Not used |
| 4.16 | Metabolomics | Specify the analytic method used (such as nuclear magnetic resonance spectroscopy or mass spectrometry). For mass spectrometry, detail which fractions were obtained (polar and/or non polar) and how these were analyzed. Provide details on metabolomics methods and platforms (e.g. derivatization, instrument type, injection type, column type and instrument settings). | STORMS | Provide details on any internal standards which may have been used as well as parameters and versions of any software or databases used. | Yes | This is provided in the methods section. |

| | | | | | | |
|---|---|---|---|---|---|---|
| 5.0 | Data sources/ measurement | For each non-microbiome variable, including the health condition, intervention, or other variable of interest, state how it was defined, how it was measured or collected, and any transformations applied to the variable prior to analysis. | MIxS: host disease status | State any sources of potential bias in measurements, for example multiple interviewers or measurement instruments, and whether these potential biases were assessed or accounted for in study design.<br><br>Use terms from a standardized ontology such as the Experimental Factor Ontology (https://www.ebi.ac.uk/efo/) to describe variables of interest in a standardized format. | Yes | Cohort and non-microbiome variables were previously described; defined measures are clearly stated in manuscript |
| 6.0 | Research design for causal inference | Discuss any potential for confounding by variables that may influence both the outcome and exposure of interest. State any variables controlled for and the rationale for controlling for them. | STORMS | For causal inference, this item refers to describing the assumptions that would be required to draw causal inferences from observational data. See Vujkovic-Cvijin, I., Sklar, J., Jiang, L. et al. Host variables confound gut microbiota studies of human disease. Nature 587, 448–454 (2020). https://doi.org/10.1038/s41586-020-2881-9 for more details on confounding in observational microbiome studies.<br><br>For example, hypothesized confounders may be controlled for by multivariable adjustment. Consider using a directed acyclic graph (DAG) to describe your causal model and justify any variables controlled for. DAGs can be made using www.dagitty.net. | NA | Cofounders and variables were not controlled prior to analysis. We aim to study real world conditions to learn the true relationship between gut microbes and OCV response in a cholera-endemic population. |

| | | | | | | |
|---|---|---|---|---|---|---|
| 6.1 | Selection bias | Discuss potential for selection or survival bias. | STORMS | Selection bias can occur when some members of the target study population are more likely to be included in the study/final analytic sample than others. Some examples include survival bias (where part of the target study population is more likely to die before they can be studied), convenience sampling (where members of the target study population are not selected at random), and loss to follow-up (when probability of dropping out is related to one of the things being studied). | NA | Bias of participants included / cohort are previously discussed in parent study |
| 7.0 | Bioinformatic and Statistical Methods | Describe any transformations to quantitative variables used in analyses (e.g. use of percentages instead of counts, normalization, rarefaction, categorization). | STORMS | If a variable is analyzed using different transformations, state rationale for the transformation and for each analyses which version of the variable is used.<br><br>In case of any complex or multistep transformations, give enumerated instructions for reproducing those transformations. | Yes | Any and all transformations of quantitative variables are described |
| 7.1 | Quality Control | Describe any methods to identify or filter low quality reads or samples. | MIxS: sequence quality check | If samples were excluded based on quality or read depth, list the criteria used, the number of samples excluded, and the final sample size after quality control. | Yes | This is provided in the methods section. |
| 7.2 | Sequence analysis | Describe any taxonomic, functional profiling, or other sequence analysis performed. | MIxS: feature prediction; similarity search method | | Yes | This is provided in the methods section. |

| | | | | | | |
|---|---|---|---|---|---|---|
| 7.3 | Statistical methods | Describe all statistical methods. | Modified STROBE | Describe any statistical tests used, exploratory data analysis performed, dimension reduction methods/unsupervised analysis, alpha/beta metrics, and/or methods for adjusting for measurement bias.<br><br>If multiple statistical methods are possible, discuss why the methods used were selected.<br><br>If a multiple hypothesis testing correction method was used, describe the type of correction used.<br><br>State which taxonomic levels are analyzed. | Yes | This is provided in the methods section and in the figure legends. |
| 7.4 | Longitudinal analysis | If the study is longitudinal, include a section that explicitly states what analysis methods were used (if any) to account for grouping of measurements by individual or patterns over time. | STORMS | | NA | |
| 7.5 | Subgroup analysis | Describe any methods used to examine subgroups and interactions. | STROBE | | NA | |
| 7.6 | Missing data | Explain how missing data were addressed. | STROBE | "Missing data" refers to participant measurements such as covariates, exposures, outcomes, or time points that should have been collected but were not, not to zeros in taxonomic abundance tables or data points not applicable to that observation. | Yes | This is described in the methods. |
| 7.7 | Sensitivity analyses | Describe any sensitivity analyses. | STROBE | | NA | |
| 7.8 | Findings | State criteria used to select findings for reporting. | STORMS | For example, false discovery rate with total number of tests, effect size threshold, significance threshold, microbes of interest. | Yes | This is described in the methods. |

| | | | | | | |
|---|---|---|---|---|---|---|
| 7.9 | Software | Cite all software (including read mapping software) and databases (including any used for taxonomic reference or annotating amplicons, if applicable) used. Include version numbers. | Modified STREGA | Installed packages, add-ons or libraries should be stated and cited in addition to the software used.<br><br>All parameters employed that differ from the default of that software/version should be provided.<br><br>This is in addition to, not a replacement for, publishing of code as outlined in the section Reproducible Research. | Yes | This is described in the methods. |
| 8.0 | Reproducible research | Make a statement about whether and how others can reproduce the reported analysis. | STORMS | Any protected information that has been excluded or provided under controlled access should be listed along with any relevant data access procedures. "On request from authors" is not sufficiently detailed; formal data access procedures and conditions should be defined.<br><br>If data are unavailable, state so clearly.<br><br>Consider using a specialized rubric for reproducible research (such as: https://mbio.asm.org/content/9/3/e00525-18.short).<br><br>Consider preregistering the study protocol (such as on osf.io or https://plos.org/open-science/preregistration/). | Yes | Methods are provided, all data is available |
| 8.1 | Raw data access | State where raw data may be accessed including demultiplexing information. | STORMS | Robust, long-term databases such as those hosted by NCBI and EBI are preferred. If using a private repository, provide rationale. | Yes | NCBI SRA database repository and MassIVE are used. |

| | | | | | | |
|---|---|---|---|---|---|---|
| 8.2 | Processed data access | State where processed data may be accessed. | STORMS | Unfiltered data should be provided.<br><br>Robust, long-term databases such as those hosted by NCBI and EBI-EMBL are preferred. Repositories like zenodo (https://zenodo.org/) or publisso (https://www.publisso.de/en/working-for-you/doi-service/)<br>can be used to provide a DOI and long-term storage for processed datasets, even those which cannot be published openly. | Yes | Data is provided as "Source Data" or in the Supplementary Data |
| 8.3 | Participant data access | State where individual participant data such as demographics and other covariates may be accessed, and how they can be matched to the microbiome data. | STORMS | If re-categorized, transformed, or otherwise derived variables were used in the analysis, these variables or code for deriving them should be provided.<br><br>Examples of how participant data can be matched to microbiome data are: using the same set of anonymized identifiers, or using different anonymized identifiers but providing a map.<br><br>Provided data should be sufficient to independently replicate the current analysis. | Yes | Data is provided as "Source Data" or in the Supplementary Data |
| 8.4 | Source code access | State where code may be accessed. | STORMS | If a standard or formalized workflow was employed, reference it here. | Yes | All souce data is available |
| 8.5 | Full results | Provide full results of all analyses, in computer-readable format, in supplementary materials. | STORMS | For example, any fold-changes, p-values, or FDR values calculated, provided as a spreadsheet.<br><br>Use a machine-readable, plain-text format such as csv or tsv. | Yes | All source data is available |
| **Results** | | | | | | |

| | | | | | | |
|---|---|---|---|---|---|---|
| 9.0 | Descriptive data | Give characteristics of study participants (e.g. dietary, demographic, clinical, social) and information on exposures and potential confounders. | STROBE | Typically reported in a table included in the paper or as a supplementary table. Indicate number of participants with missing data for each variable of interest.<br><br>This includes environmental and lifestyle factors that may affect the relationship between the microbiome and the condition of interest. Participant diet and medication use should be summarized, if known.<br><br>At minimum, age and sex of all participants should be summarized. | Yes | |
| 10.0 | Microbiome data | Report descriptive findings for microbiome analyses with all applicable outcomes and covariates. | STORMS | This includes measures of diversity as well as relative abundances. These descriptive findings should be reported both for the sample overall and for individual groups. | Yes | |
| 10.1 | Taxonomy | Identify taxonomy using standardized taxon classifications that are sufficient to uniquely identify taxa. | STORMS | If not using full taxonomic hierarchy, make sure it is clear whether names stated are species, genera, family, etc.<br><br>Italicize genus/species pairs. Consult journal guidelines or standardized references on taxonomic nomenclature. For instance, https://wwwnc.cdc.gov/eid/page/scientific-nomenclature | Yes | |
| 10.2 | Differential abundance | Report results of differential abundance analysis by the variable of interest and (if applicable) by time, clearly indicating the direction of change and total number of taxa tested. | STORMS | If there are more than two groups, include omnibus (multigroup) test results if applicable to the research question.<br><br>If applicable, reported effect sizes should include a measure of uncertainty such as the confidence interval. | Yes | |
| 10.3 | Other data types | Report other data analyzed--e.g. metabolic function, functional potential, MAG assembly, and RNAseq. | STORMS | | Yes | |

| | | | | | | |
|---|---|---|---|---|---|---|
| 10.4 | Other statistical analysis | Report any statistical data analysis not covered above. | STORMS | This could include subgroup analysis, sensitivity analyses, and cluster analysis.<br><br>Visualizations should be easily interpretable and colorblind-friendly. The caption and/or main text should provide a detailed description of visualizations for visually-impaired readers. | Yes | |
| **Discussion** | | | | | | |
| 11.0 | Key results | Summarise key results with reference to study objectives | STROBE | | Yes | |
| 12.0 | Interpretation | Give a cautious overall interpretation of results considering objectives, limitations, multiplicity of analyses, results from similar studies, and other relevant evidence. | STROBE | Define or clarify any subjective terms such as "dominant," "dysbiosis," and similar words used in interpretation of results.<br><br>When interpreting the findings, consider how the interpretation of the findings may be summarized or quoted for the general public such as in press releases or news articles.<br><br>If causal language is used in the interpretation (such as "alters," "affects," "results in," "causes," or "impacts"), assumptions made for causal inference should be explicitly stated as part of 6.0 and 13.0.<br><br>Distinguish between function potential (ie inferred from metagenomics) and observed activity (ie metatranscriptomic, metabolomic, proteomic) if discussing microbial function. | Yes | |
| 13.0 | Limitations | Discuss limitations of the study, taking into account sources of potential bias or imprecision. | STROBE | Also consider limitations resulting from the methods (especially novel methods), the study design, and the sample size. | Yes | |
| 13.1 | Bias | Discuss any potential for bias to influence study findings. | STORMS | May include sampling method, representativeness of study participants, or potential confounding. | Yes | |

| | | | | | | |
|---|---|---|---|---|---|---|
| 13.2 | Generalizability | Discuss the generalisability (external validity) of the study results | STROBE | To what populations or other settings do you expect the conclusions to generalize? | Yes | |
| 14.0 | Ongoing/future work | Describe potential future research or ongoing research based on the study's findings. | STORMS | | Yes | |
| **Other information** | | | | | | |
| 15.0 | Funding | Give the source of funding and the role of the funders for the present study and, if applicable, for the original study on which the present article is based | STROBE | | Yes | |
| 15.1 | Acknowledgements | Include acknowledgements of those who contributed to the research but did not meet critera for authorship. | STORMS | For general guidelines on authorship, see http://www.icmje.org and https://www.elsevier.com/authors/journal-authors/policies-and-ethics/credit-author-statement | Yes | |
| 15.2 | Conflicts of Interest | Include a conflicts of interest statement. | STORMS | | Yes | |
| 16.0 | Supplements | Indicate where supplements may be accessed and what materials they contain. | STORMS | | Yes | |
| 17.0 | Supplementary data | Provide supplementary data files of results with for all taxa and all outcome variables analyzed. Indicate the taxonomic level of all taxa. | STORMS | Depending on the analysis performed, examples of the supplemental results included could be mean relative abundance, differential abundance, raw p-value, multiple hypothesis testing-adjusted p-values, and standard error.<br><br>All discussed taxa should include the taxonomic level (e.g. class, order, genus). | Yes | Data is provided as "Source Data" or in the Supplementary Data |

**PARTNERS HUMAN RESEARCH COMMITTEE**
**PROTOCOL SUMMARY**

**Answer all questions accurately and completely in order to provide the PHRC with the relevant information to assess the risk-benefit ratio for the study. Do not leave sections blank.**

## PRINCIPAL/OVERALL INVESTIGATOR
Regina C. LaRocque

## PROTOCOL TITLE
Immune responses to cholera vaccination in Bangladesh

## FUNDING
HHMI

## VERSION DATE
4/15/15

## SPECIFIC AIMS
Concisely state the objectives of the study and the hypothesis being tested.

**Hypothesis**: We hypothesize that the oral cholera vaccine activates distinct mucosal innate immune pathways as well as adaptive responses to as yet unidentified protein antigens found in V. cholerae strains which may contribute to immunity.

**Objective**: We propose to obtain serial blood samples from 50 adults and 50 children who are vaccinated with the killed oral cholera vaccine, Dukoral®, and to measure innate and adaptive immune responses to both previously characterized and uncharacterized V. cholerae antigens.

## BACKGROUND AND SIGNIFICANCE
Provide a brief paragraph summarizing prior experience important for understanding the proposed study and procedures.

Vibrio cholerae causes 3 to 5 million cases of cholera and over 100,000 deaths annually. The increasing burden of cholera suggests that more aggressive approaches to preventing cholera, including the use of oral cholera vaccines, are needed. Because V. cholerae is a non-invasive pathogen, it is also an excellent model for the study of immunity at the mucosal surface. Identification of these signalling pathways activated oral cholera vaccines may enhance our understanding of the mechanisms which lead to long-lasting mucosal immunity. Oral vaccines have been developed to protect against cholera. Dukoral (Crucell, Sweden) the whole cell inactivated (O1 serogroup) cholera vaccine containing recombinant cholera toxin B subunit (rCTB) was developed over 30 years ago and WHO

prequalified and licensed in over 60 countries including Bangladesh. While the immunogenicity of the Dukoral vaccine has been studied, including primarily immune responses to rCTB and to the V. cholerae O1 lipopolysaccharide antigen, Dukoral as a killed oral vaccine is a complex mixture of different antigens and other components which may have adjuvant properties. We would therefore like to use Dukoral for the present study to better understand the innate and adaptive responses to this killed oral vaccine, including the identification of novel antigens using a protein microarray (in the final stages of production). This knowledge may ultimately be applied to other vaccines and to understand the generation of new cholera vaccines, or other mucosal vaccines or adjuvants for other enteric diseases.

## RESEARCH DESIGN AND METHODS

Briefly describe study design and anticipated enrollment, i.e., number of subjects to be enrolled by researchers study-wide and by Partners researchers. Provide a brief summary of the eligibility criteria (for example, age range, gender, medical condition). Include any local site restrictions, for example, “Enrollment at Partners will be limited to adults although the sponsor’s protocol is open to both children and adults.”

### Study Setting

This study will be conducted at the International Centre for Diarrheoal Disease Research, Bangladesh by a team of experienced investigators in the field, in a cholera endemic area where the vaccine has been extensively tested, is licensed and used commercially.

### Study Design

We propose to enroll 50 adults and 50 children in a study of immune response to oral cholera vaccine. All the enrolled adults and children will receive Dukoral and undergo blood tests at four time points relative to the first dose of vaccine. Blood samples will be collected at four time points (On day -14, 2, 7 and 44 post vaccination).

### Vaccine

Dukoral, a killed whole cell oral cholera vaccine that includes recombinant cholera toxin B subunit (WC-rCTB). It is prequalified by the World Health Organization and one of the oral cholera vaccines recommended in cholera endemic countries. It is also used for travelers to cholera endemic countries for protection against cholera and ETEC diarrhea. It contains V. cholerae O1 Inaba classic strain, heat inactivated (ca. 31.25 x109 vibrios), V. cholerae O1 Inaba El Tor strain, formalin inactivated (ca. 31.25 x 109 vibrios), V. cholerae O1 Ogawa classical strain, heat inactivated (ca. 31.25 x 109 vibrios), V. cholerae O1 Ogawa classical strain, formalin inactivated (ca. 31.25 x 109 vibrios) and recombinant cholera toxin B subunit (rCTB) 1 mg. The vaccine will be stored at 2-8 °C at the ICDDR,B hospital pharmacy.

To date, Dukoral has been used in several large studies in epidemic and endemic cholera settings in Africa and Asia and has been shown to be safe, immunogenic, and protective.

**Study population**

The study population will include 50 adult males or non-pregnant females, 18-65 years of age, as well as 50 children 2-17 years of age residing in and around Dhaka city.

**Eligibility Criteria**

Inclusion Criteria:

- Male or non pregnant females
- Age 2 to 65 years, inclusive
- Provision of informed consent for enrollment in study by patient/parent or guardian of children, as well as assent for children age 11 to 17 years, inclusive.

Exclusion Criteria:

- Prior history of oral cholera vaccination
- Pregnancy (pregnancy will be excluded by urine HCG testing at day 0 and day 14 before each vaccine administration)
- Unable to give informed consent

Individuals that are eligible for oral cholera vaccination will be invited to participate in this research study, and will be recruited by community health workers prior to study start state. Trained research staff will review inclusion/exclusion criteria with potential participants, and for those who agree to participate, informed consent will be obtained and participant information will be recorded in the study registry.

**Study Timeline**

Individuals presenting to an outpatient clinic that are eligible for oral cholera vaccination will be invited to participate in this research study. Trained research staff will review inclusion/exclusion criteria with potential participants, and for those who agree to participate, informed consent will be obtained. Study physicians will perform an initial assessment and study nurse will collect blood and stool at various time points. Study participants will come to the icddr,b or the icddr,b field site in Mirpur for vaccination and for blood collection, which will take place in a dedicated study room. Questionnaires will be filled out upon enrollment and at each study visit.

The killed oral cholera vaccine, Dukoral, will be administered in two doses 14 days apart by the study personnel in accordance with the manufacturer's instructions.

Venous blood samples will be obtained using hygienic techniques and disposable supplies, to minimize the risk of infection. The volume of blood drawn will be age dependent:

Ages 2-5 years: 3 -5mL (approximately ½-1 teaspoon)
Ages 6-10 years: 5 mL (approximately 1 teaspoon)
Ages 11-17: 10 ml (approximately 2 teaspoons)
Ages 18 and older: 10-15 ml (approximately 2-3 teaspoons)

Blood will be collected on day -14, day 2, day 7 and day 44.

Collection of stool: A stool sample will be taken at baseline from all participants, additional stool samples may be collected if feasible at the same time points as blood collection for all participants.

Enrolment: We would aim to enroll all participants over a four year period.

Vaccine administration: Individuals will receive 2 doses of oral cholera vaccine, Dukoral (Crucell). Vaccine, stored at between 2-8 degrees Celsius, will be administered orally on day 0 and a second dose of the vaccine will be provided as recommended 2 weeks after the initial dose per manufacturer's instructions.

**Specimen Processing**
Blood and stool specimens will be processed, de-identified of personal information, and assigned a study number. Blood specimens will be exported, in accordance with International Air Transport Association (IATA) and Public Health Service regulations, to Massachusetts General Hospital to the laboratories of Dr. Harris and LaRocque for immunologic analysis.

Briefly describe study procedures. Include any local site restrictions, for example, "Subjects enrolled at Partners will not participate in the pharmacokinetic portion of the study." Describe study endpoints.

### Data Collection

A registry will be created that contains enrollment information on the study subjects including name, gender, age, address, contact phone number. This registry will also document their vaccination dates and batch numbers for the vaccine doses received. A study number will be assigned to each subject to link patient information. The study registry will be secured in a locked filing cabinet to which only the research personnel for this study will have access.

The Vaccinee Enrollment Form survey will be administered. Information will be uploaded to a secure (encrypted/password protected), web-based database. All data will be maintained in a secured data management system with password protection, electronic signatures, and an audit trail. Local staff will be trained regarding confidentiality in patient care as part of their routine activities and on-site study staff will be trained regarding research ethics and confidentiality according to international IRB standards. Since study IDs are used, participant names are

delinked and will not be available to study staff once the electronic database is created.

No PHI will be disclosed outside other than to icddr,b investigators.

**Specimen Collection, Processing, Storage and Shipment**

*Stool collection*. Stool specimens will be collected from subjects after defecation and placing a small amount of stool into a sterile specimen container provided by the study staff.

*Venipuncture.* Collection of blood will only be performed by trained phlebotomists using disposable equipment and universal precautions. Venous blood samples will be obtained using hygienic techniques and disposable supplies, to minimize the risk of infection. The volume of blood drawn at each time point will be:

Ages 2-5 years: 3 -5mL (approximately ½-1 teaspoon)
Ages 6-10 years: 5 mL (approximately 1 teaspoon)
Ages 11-17: 10 ml (approximately 2 teaspoons)
Ages 18 and older: 10-15 ml (approximately 2-3 teaspoons)

Even with the repeated blood draws, this volume is much lower than maximum allowable under Partners Health Care Blood Sampling guidelines.

*Processing of samples.* Venous blood will be deposited in purple top tubes and/or Cell Preparation Tubes (CPT). Peripheral blood mononuclear cells (PBMCs) will be separated from plasma using gradient centrifugation. All plasma samples will be stored at -80° C. Some PBMCs may be used immediately for immunologic assays on site (see below), while others will be frozen and transferred to a liquid nitrogen filled dry-shipper. Stool samples will be subject to routine culture and microscopy may be bioprotected with the addition of 15% glycerol then frozen at -80° C. Once all frozen, stored specimens from a single time point have been collected, they will be immediately shipped on dry ice to Boston, MA for analysis.

*Shipping of samples*. All samples will be shipped to Massachusetts General Hospital in accordance with International Air Transport Association (IATA) and Public Health Service regulations.

*Processing of blood specimens.* No more than 15 ml of blood will be drawn at any time point. Processing of the blood sample to test for *V. cholerae* specific antibodies and lymphocytes will occur at the iccdr,b Immunology laboratory under the direction of the investigators.

*Immune Responses*. Vibriocidal antibody assays will be performed with standardized methodology. Plasma samples will be tested for antibody responses to LPS and CtxB, or other antigens using kinetic. Antibody in lymphocyte supernatant (ALS) and assays for antigen-specific memory B cell will also be conducted. We will probing a *V. cholerae* protein array using: (1) plasma, (2) antibodies in lymphocyte secretions (ALS fluid), and (3) cell culture supernatants from memory B cells.

*Non-immunologic analysis*. Blood group designation will be assigned to each participant by backtyping from plasma samples according to standard methods. Levels of selected micronutrients including retinol levels may be measured in plasma at the Harvard Nutrition core facility. Stool specimens will be screened for geohelminths and other selected enteric organisms.

*Publications*. Results of the study will be shared locally at the icddr,b and MGH, international conferences and submitted to a peer-reviewed journals for publication.

**ICDDR,B Ethics Review Committee Approval.** This protocol was approved by the icddr,b ethics review committee in December 2014 (PR-14086).

For studies involving treatment or diagnosis, provide information about standard of care at Partners (e.g., BWH, MGH) and indicate how the study procedures differ from standard care. Provide information on available alternative treatments, procedures, or methods of diagnosis.

There is no standard related to cholera vaccination at MGH as cholera is not endemic and the vaccine is not available. Participants will receive Dukoral, an oral cholera vaccine, that is WHO prequalified and licensed in Bangladesh. While available this vaccine is rarely used because there is no national cholera vaccination program at present in Bangladesh although this is being evaluated by the present Goverment of Bangladesh. Participation in this study will not affect an individual's access to care, diagnosis and treatment for cholera or other medical conditions.

Describe how risks to subjects are minimized, for example, by using procedures which are consistent with sound research design and which do not unnecessarily expose subjects to risk or by using procedures already being performed on the subject for diagnostic or treatment purposes.

Risks associated with the study are small and include the risk of discomfort from a blood draw, risk of adverse effects from the vaccine, inconvenience of return trips to the clinic for blood draw, and/or risk of breach of protected health information. Measures will be taken to ensure minimal risk to the participant.

(i) Access to protected health information (PHI) will be limited to key study personnel. As soon as enrollment is complete, PHI will be separated from participant specimens and a study number assigned. The Massachusetts General Hospital investigators will not have access to PHI.

(ii) The study doctors are trained employees of the icddr,b. They will receive specific training regarding issues of confidentiality before their participation in the study.

(iii) Confidentiality is considered of chief importance in training members of the study staff.

(iv) Subjects will be encouraged not to participate if they do not feel comfortable doing so and will be advised that if they do not participate,

their medical care will not be influenced in any way. Interviews will take place in a private room.

Describe explicitly the methods for ensuring the safety of subjects. Provide objective criteria for removing a subject from the study, for example, objective criteria for worsening disease/lack of improvement and/or unacceptable adverse events. The inclusion of objective drop criteria is especially important in studies designed with placebo control groups.

Dukoral is prequalified by the World Health Organization and has been safely administered to hundreds of thousands of individuals without serious associated adverse events. After each dose of the vaccine, the participants will be monitored for 30 minutes in the clinic prior to discharge. Participants will be instructed to notify study doctor if they have any problems and symptom histories will be solicited from participants at each visit. Any adverse events will be addressed by medical staff.

## FORESEEABLE RISKS AND DISCOMFORTS

Provide a brief description of any foreseeable risks and discomforts to subjects. Include those related to drugs/devices/procedures being studied and/or administered/performed solely for research purposes. In addition, include psychosocial risks, and risks related to privacy and confidentiality. When applicable, describe risks to a developing fetus or nursing infant.

The risks associated with participation in this study are minimal. The most important forseeable risk associated with participation is a breach in confidentiality, if protected health information is accessed by others not part of the study or medical team. This risk will be minimized by procedures to ensure data confidentiality and security, described in the Research Design/Methods and Privacy/Confidentiality sections.

A second potential risk to participation is discomfort from blood draws, but volume of blood drawn will be minimized and only trained phlebotomists will perform the procedure using universal precautions.

## EXPECTED BENEFITS

Describe both the expected benefits to individual subjects participating in the research and the importance of the knowledge that may reasonably be expected to result from the study. Provide a brief, realistic summary of potential benefits to subjects, for example, "It is hoped that the treatment will result in a partial reduction in tumor size in at least 25% of the enrolled subjects." Indicate how the results of the study will benefit future patients with the disease/condition being studied and/or society, e.g., through increased knowledge of human physiology or behavior, improved safety, or technological advances.

The individuals participating in the research will receive oral cholera vaccine as part of the study. At this time, the wider introduction of any oral cholera vaccine in the larger community is still uncertain. Therefore, it is possible that individuals participating in this study, as well as their family members, may receive the vaccine, while others in their local community may not. Alternatively, participants may benefit from receiving the vaccine sooner than if they were in the community if a mass vaccination campaign does take place. Otherwise, there is no specific benefit to the individual subject in this study.

**EQUITABLE SELECTION OF SUBJECTS**

The risks and benefits of the research must be fairly distributed among the populations that stand to benefit from it. No group of persons, for example, men, women, pregnant women, children, and minorities, should be categorically excluded from the research without a good scientific or ethical reason to do so. Please provide the basis for concluding that the study population is representative of the population that stands to potentially benefit from this research.

Pregnant women are not eligible for the vaccine because of a lack of safety data on the use of the vaccine in pregnant women.

When people who do not speak English are excluded from participation in the research, provide the scientific rationale for doing so. Individuals who do not speak English should not be denied participation in research simply because it is inconvenient to translate the consent form in different languages and to have an interpreter present.

This is not applicable. All subjects are most likely be native Bangla speakers.

For guidance, refer to the following Partners policy:

Obtaining and Documenting Informed Consent of Subjects who do not Speak English
**https://partnershealthcare-public.sharepoint.com/ClinicalResearch/Non-English_Speaking_Subjects.1.10.pdf**

**RECRUITMENT PROCEDURES**

Explain in detail the specific methodology that will be used to recruit subjects. Specifically address how, when, where and by whom subjects will be identified and approached about participation. Include any specific recruitment methods used to enhance recruitment of women and minorities.

The study doctor or staff will invite potential participants who are attending for clinical services in at the iccdr,b or its field sites to enroll in the study. Individuals will be enrolled sequentially until a pre-determined number of subjects is accrued. The study doctor or nurse will explain the study to participants, invite them to participate, and conduct informed consent (as described below).

Provide details of remuneration, when applicable. Even when subjects may derive medical benefit from participation, it is often the case that extra hospital visits, meals at the hospital, parking fees or other inconveniences will result in additional out-of-pocket expenses related to study participation. Investigators may wish to consider providing reimbursement for such expenses when funding is available

*Remuneration*. The costs associated with participation in this study include the time and transport costs for coming for blood draws on day -14, day 2, day 7 and day 44, and vaccination on days 0 and 14, a total of 6 visits. Participants may also incur costs for eating meals away from home, childcare on the days of their study visits, and others may live at a distance from the clinic. Participants will be compensated 300-500 Taka (approximately US$5-8) for their transport and other related costs for each study visit. This amount is a standard compensation that has been used in previous local research studies.

For guidance, refer to the following Partners policies:

Recruitment of Research Subjects
https://partnershealthcare-public.sharepoint.com/ClinicalResearch/Recruitment_Of_Research_Subjects.pdf

Guidelines for Advertisements for Recruiting Subjects
https://partnershealthcare-public.sharepoint.com/ClinicalResearch/Guidelines_For_Advertisements.1.11.pdf

Remuneration for Research Subjects
https://partnershealthcare-public.sharepoint.com/ClinicalResearch/Remuneration_for_Research_Subjects.pdf

## CONSENT PROCEDURES

Explain in detail how, when, where, and by whom consent is obtained, and the timing of consent (i.e., how long subjects will be given to consider participation). For most studies involving more than minimal risk and all studies involving investigational drugs/devices, a licensed physician investigator must obtain informed consent. When subjects are to be enrolled from among the investigators' own patients, describe how the potential for coercion will be avoided.

### Informed Consent

Prior to enrolling in the study, informed consent shall be documented by the use of a written consent form and signed by the subject. At the time of study enrollment, a dedicated study doctor or nurse will give the subject a consent form describing in detail the study procedures. The informed consent process will be conducted in the local language of Bangla. The study worker shall give the subject adequate opportunity to read the consent form before it is signed. It will be emphasized that participation in this study is strictly voluntary and non-participation will not affect the right to care or services to which the potential participant would otherwise be entitled. If the subject agrees to participate and provide consent, the study worker

will ask the subject to sign two copies of the informed consent document. The study worker will give one of these two signed copies to the subject.

At the time the study worker seeks informed consent, s/he will ask the eligible subject if the subject is literate. If the eligible subject reports he or she is not literate, then the study worker will request that a witness be present while the study worker reads and explains the study and what participation will entail. If the eligible subject accepts to participate, he or she will make a mark on the signature line of the consent form. The witness will also sign and date the form, if the witness is confident that the subject has understood the explanation and is participating willingly. In addition, the witness will complete the date line for the subject.

Consent will be sought from all individuals prior to initiation of study activities.

NOTE: When subjects are unable to give consent due to age (minors) or impaired decision-making capacity, complete the forms for Research Involving Children as Subjects of Research and/or Research Involving Individuals with Impaired Decision-making Capacity, available on the New Submissions page on the PHRC website:
**https://partnershealthcare.sharepoint.com/sites/phrmApply/aieipa/irb**

For guidance, refer to the following Partners policy:
Informed Consent of Research Subjects:
**https://partnershealthcare-public.sharepoint.com/ClinicalResearch/Informed_Consent_of_Research_Subjects.pdf**

## DATA AND SAFETY MONITORING

Describe the plan for monitoring the data to ensure the safety of subjects. The plan should include a brief description of (1) the safety and/or efficacy data that will be reviewed; (2) the planned frequency of review; and (3) who will be responsible for this review and for determining whether the research should be altered or stopped. Include a brief description of any stopping rules for the study, when appropriate. Depending upon the risk, size and complexity of the study, the investigator, an expert group, an independent Data and Safety Monitoring Board (DSMB) or others might be assigned primary responsibility for this monitoring activity.

NOTE: Regardless of data and safety monitoring plans by the sponsor or others, the principal investigator is ultimately responsible for protecting the rights, safety, and welfare of subjects under his/her care.

A Research Coordinator will monitor all incoming data for quality and completeness. The PI will review the study procedures at regular (bi-weekly) meetings and will ensure the accuracy, quality, and completeness of the records. A physician Investigator will review all laboratory results and the Principal Investigator will review those that are clinically significant. Should an adverse event relating to

venipuncture or vaccine administration occur, the study principal investigator will be notified within 24 hours.

Describe the plan to be followed by the Principal Investigator/study staff for review of adverse events experienced by subjects under his/her care, and when applicable, for review of sponsor safety reports and DSMB reports. Describe the plan for reporting adverse events to the sponsor and the Partners' IRB and, when applicable, for submitting sponsor safety reports and DSMB reports to the Partners' IRBs. When the investigator is also the sponsor of the IND/IDE, include the plan for reporting of adverse events to the FDA and, when applicable, to investigators at other sites.

NOTE: In addition to the adverse event reporting requirements of the sponsor, the principal investigator must follow the Partners Human Research Committee guidelines for Adverse Event Reporting

Any unanticipated problems or events will be reported to the icddr,b Principal Investigator (Dr. Firdausi Qadri) and the Partners Principal Investigator (LaRocque) by study staff and by the PI to the Partners IRB.

## MONITORING AND QUALITY ASSURANCE

Describe the plan to be followed by the principal investigator/study staff to monitor and assure the validity and integrity of the data and adherence to the IRB-approved protocol. Specify who will be responsible for monitoring, and the planned frequency of monitoring. For example, specify who will review the accuracy and completeness of case report form entries, source documents, and informed consent.

NOTE: Regardless of monitoring plans by the sponsor or others, the principal investigator is ultimately responsible for ensuring that the study is conducted at his/her investigative site in accordance with the IRB-approved protocol, and applicable regulations and requirements of the IRB.

The PI will work closely with the study doctor / nurse to ensure the validity of the data. All staff have completed IRB training modules.

For guidance, refer to the following Partners policies:

Data and Safety Monitoring Plans and Quality Assurance
**https://partnershealthcare-public.sharepoint.com/ClinicalResearch/DSMP_in_Human_Subjects_Research.pdf**

Reporting Unanticipated Problems (including Adverse Events)
**https://partnershealthcare-public.sharepoint.com/ClinicalResearch/Reporting_Unanticipated_Problems_including_Adverse_Events.pdf**

**PRIVACY AND CONFIDENTIALITY**

Describe methods used to protect the privacy of subjects and maintain confidentiality of data collected. This typically includes such practices as substituting codes for names and/or medical record numbers; removing face sheets or other identifiers from completed surveys/questionnaires; proper disposal of printed computer data; limited access to study data; use of password-protected computer databases; training for research staff on the importance of confidentiality of data, and storing research records in a secure location.

NOTE: Additional measures, such as obtaining a Certificate of Confidentiality, should be considered and are strongly encouraged when the research involves the collection of sensitive data, such as sexual, criminal or illegal behaviors.

Clinical Data: Pertinent clinical data required from cases will be captured by the study nurse or doctor in the registry.

Protected health information (PHI), along with details of the patient's clinical status, are initially entered into the registry, and a study number is generated. After enrollment is complete, the data will be entered into an electronic database; PHI will be separated and maintained in a separate locked file to which only the PI and the Research team have access.

**SENDING SPECIMENS/DATA TO RESEARCH COLLABORATORS OUTSIDE PARTNERS**

Specimens or data collected by Partners investigators will be sent to research collaborators outside Partners, indicate to whom specimens/data will be sent, what information will be sent, and whether the specimens/data will contain identifiers that could be used by the outside collaborators to link the specimens/data to individual subjects.

De-identified specimens (stool, plasma and/or peripheral mononuclear cells) may be shipped to collaborators at Emory University, Duke University, and/or the Broad Institute of MIT/Harvard, or other academic collaborators for specific immunologic assays. Any samples sent will be under the auspice of a Partners approved Material Transfer Agreement. All samples sent will be stripped of all personal identifiers. (No personal identifiers are available to Partners Investigators either).

Specifically address whether specimens/data will be stored at collaborating sites outside Partners for future use not described in the protocol. Include whether subjects can withdraw their specimens/data, and how they would do so. When appropriate, submit documentation of IRB approval from the recipient institution.

Specimens will not be stored at the collaborating site.

**RECEIVING SPECIMENS/DATA FROM RESEARCH COLLABORATORS OUTSIDE PARTNERS**

When specimens or data collected by research collaborators outside Partners will be sent to Partners investigators, indicate from where the specimens/data will be obtained and whether the specimens/data will contain identifiers that could be used by Partners investigators to link the specimens/data to individual subjects. When appropriate, submit documentation of IRB approval and a copy of the IRB-approved consent form from the institution where the specimens/data were collected.

The protocol has been approved by the icddr,b Ethics Review Committee.
