# Supplementary material for: Gut bacteria-derived sphingolipids alter innate immune responses to oral cholera vaccine antigens
Source: Nat Commun. 2025 Dec 12;17:638. doi: 10.1038/s41467-025-67388-y (PMC12816596; doi:10.1038/s41467-025-67388-y)
Supplement: Supplementary file 9 — Reporting Summary [file 41467_2025_67388_MOESM9_ESM.pdf]

nature portfolio

Corresponding author(s): Ana A. Weil

Last updated by author(s): Nov 18, 2025

Reporting Summary

Nature Portfolio wishes to improve the reproducibility of the work that we publish. This form provides structure for consistency and transparency in reporting. For further information on Nature Portfolio policies, see our Editorial Policies and the Editorial Policy Checklist.

Statistics

For all statistical analyses, confirm that the following items are present in the figure legend, table legend, main text, or Methods section.

n/a	Confirmed	
☐	☒	The exact sample size (*n*) for each experimental group/condition, given as a discrete number and unit of measurement
☒	☐	A statement on whether measurements were taken from distinct samples or whether the same sample was measured repeatedly
☐	☒	The statistical test(s) used AND whether they are one- or two-sided<br>*Only common tests should be described solely by name; describe more complex techniques in the Methods section.*
☐	☒	A description of all covariates tested
☐	☒	A description of any assumptions or corrections, such as tests of normality and adjustment for multiple comparisons
☐	☒	A full description of the statistical parameters including central tendency (e.g. means) or other basic estimates (e.g. regression coefficient) AND variation (e.g. standard deviation) or associated estimates of uncertainty (e.g. confidence intervals)
☒	☐	For null hypothesis testing, the test statistic (e.g. *F*, *t*, *r*) with confidence intervals, effect sizes, degrees of freedom and *P* value noted<br>*Give P values as exact values whenever suitable.*
☒	☐	For Bayesian analysis, information on the choice of priors and Markov chain Monte Carlo settings
☒	☐	For hierarchical and complex designs, identification of the appropriate level for tests and full reporting of outcomes
☒	☐	Estimates of effect sizes (e.g. Cohen's *d*, Pearson's *r*), indicating how they were calculated

Our web collection on statistics for biologists contains articles on many of the points above.

Software and code

Policy information about availability of computer code

Data collection	No software was used for data collection
Data analysis	All software and custom algorithms with versions or data accessed are listed in the manuscript methods.<br>For processing the microbiome sequencing data into co-abundant gene groups (CAGs), fastq files were processed with geneshot (v0.9) and coding sequences were annotated using DIAMOND v0.9.10 (NCBI RefSeq accessed 01/22/2020) and functional annotations with eggNOG-mapper v2 (data accessed 05/22/2024) and NCBI blastp (data accessed 05/22/2024). CAG alignment to bacterial genomes was performed with DIAMOND via the AMGMA workflow (https://www.ncbi.nlm.nih.gov/genome/browse#!/prokaryotes/ and https://github.com/fredhutch/amgma).<br>The scripts for the priority score equation is available on GitHub (https://github.com/letsgetthisfred/Dukoral_gene_level_analysis/blob/main/Generic%20Code%20for%20priority%20score%20and%20geneshot%20output.R).<br>For processing the microbiome sequencing data for taxaonomic groupings, MetaPhlAn 2 was used with diversity measures calculated using divnet (v3.6) in R (v4.0.2).<br>For spt gene quantification, code geneshot_extract_gene_abund.py and is available here (https://gist.github.com/sminot/cebfbd84d57406b5b41b2eebffb1789f).<br>For spt gene annotation, EggNog v5.0 was used.<br>For RNA-sequencing, the RNA snakemake pipeline SEAsnake v1.1 was used (https://zenodo.org/records/11646755). Human reads were aligned to GRCh38 (release 108) and analyzed in R (v4.3.2) with kmFit (https://github.com/BIGslu/kimma). Enrichment pathways were determined using SEARchways (https://github.com/BIGslu/SEARchways) with the Broad Molecular Signatures Database.

For manuscripts utilizing custom algorithms or software that are central to the research but not yet described in published literature, software must be made available to editors and reviewers. We strongly encourage code deposition in a community repository (e.g. GitHub). See the Nature Portfolio guidelines for submitting code & software for further information.

Data

Policy information about availability of data

All manuscripts must include a data availability statement. This statement should provide the following information, where applicable:

- Accession codes, unique identifiers, or web links for publicly available datasets
- A description of any restrictions on data availability
- For clinical datasets or third party data, please ensure that the statement adheres to our policy

The whole-genome sequencing of the microbiome data generated in this study have been deposited in the SRA database under accession code PRJNA782606 [https://www.ncbi.nlm.nih.gov/bioproject/PRJNA782606]. Metadata for participant samples is provided as Supplementary Data 6. The RNA sequencing data of the THP-1-derived macrophages generated for this study have been deposited in the SRA database under accession code PRJNA1170288 [https://www.ncbi.nlm.nih.gov/bioproject/PRJNA1170288]. The targeted quantitative mass-spectrometry based lipidomics data of the fecal samples generated in this study have been deposited in the MassIVE repository under accession code MSV000099936 [https://doi.org/doi:10.25345/C54J0B99S]. The lipidomics data on the Bacteroides xylanisolvens culture generated in this study have been deposited in the MassIVE repository under MSV000099874 [https://doi.org/doi:10.25345/C58K7592K]. Metadata for the lipidomics data is provided as Supplementary Data 7.

Research involving human participants, their data, or biological material

Policy information about studies with human participants or human data. See also policy information about sex, gender (identity/presentation), and sexual orientation and race, ethnicity and racism.

Reporting on sex and gender	Data includes both male and female sex. Sex was determined by self-reporting. Consent for sharing of individual-level data was granted. Overall, 34 males and 55 females were included in our study. Sex-based analyses are included in our study.
Reporting on race, ethnicity, or other socially relevant groupings	Our study population was recruited in Dhaka, Bangladesh. No additional information on race, ethnicity, or other socially relevant grouping was collected.
Population characteristics	Our study population age ranged from 2-46 years of age.
Recruitment	Participants were recruited through announcements from community leaders. Participants were excluded if <2 years of age, due to potential bias of breastfeeding on the microbiome and maternal immunity or >60 years of age due to possible comorbidities. Participants were also excluded if he/she experienced diarrhea or had antibiotics within the past week or previously received an oral cholera vaccine.
Ethics oversight	Study was approved by ethical review committee at the International Centre for Diarrheal Disease Research, Bangaldesh (icddrb). The University of Washington, where the data was analyzed, found the study exempt from ethical review as a human study due to the de-identified samples.

Note that full information on the approval of the study protocol must also be provided in the manuscript.

Field-specific reporting

Please select the one below that is the best fit for your research. If you are not sure, read the appropriate sections before making your selection.

☒ Life sciences ☐ Behavioural & social sciences ☐ Ecological, evolutionary & environmental sciences

For a reference copy of the document with all sections, see nature.com/documents/nr-reporting-summary-flat.pdf

Life sciences study design

All studies must disclose on these points even when the disclosure is negative.

Sample size	No sample-size calculations were performed as this was a convenience sample drawn from a vaccine efficacy trial.
Data exclusions	Participant samples were excluded if memory B cell data through ELISPOT was not collected. No other data was excluded.
Replication	In vitro analyses performed with THP-1 cell and bacterial lipid fractions were performed with indicated replicates in the figures. At least 2 independent experiments were performed for each assay for reproducibility. RNA sequencing was performed on technical triplicates of THP-1 cells.<br>DNA sequencing was not performed in replicates due to rarity of samples, limited DNA, and preservation of samples for further work.
Randomization	Randomization was not relevant to our study as all participants received the oral cholera vaccine.
Blinding	Blinding was not relevant to our study as all participants received the oral cholera vaccine.

Reporting for specific materials, systems and methods

We require information from authors about some types of materials, experimental systems and methods used in many studies. Here, indicate whether each material, system or method listed is relevant to your study. If you are not sure if a list item applies to your research, read the appropriate section before selecting a response.

Materials & experimental systems

n/a	Involved in the study
☒	☐ Antibodies
☐	☒ Eukaryotic cell lines
☒	☐ Palaeontology and archaeology
☒	☐ Animals and other organisms
☐	☒ Clinical data
☒	☐ Dual use research of concern
☒	☐ Plants

Methods

n/a	Involved in the study
☒	☐ ChIP-seq
☒	☐ Flow cytometry
☒	☐ MRI-based neuroimaging

Eukaryotic cell lines

Policy information about cell lines and Sex and Gender in Research

Cell line source(s)	THP-1 cell line used in study was acquired from ATCC (TIB-202) as an immortalized cell lines. THP-1s are monocytes isolated from peripheral blood of an acute monocytic leukemia patient (male, age 1 year). PBMCs used in the study were acquired from Bloodworks Northwest (Washington, USA). PBMC donor #1 = 31 yr female, #2 = 25 yr male, #3 = 36 yr female, and #4 = 35 yr male.
Authentication	THP-1 cells were authenticated by ATCC. All PBMCs come with a certificate of analysis
Mycoplasma contamination	THP-1 cells tested negative for mycoplasma contamination.
Commonly misidentified lines (See ICLAC register)	*Name any commonly misidentified cell lines used in the study and provide a rationale for their use.*

Clinical data

Policy information about clinical studies

All manuscripts should comply with the ICMJE guidelines for publication of clinical research and a completed CONSORT checklist must be included with all submissions.

Clinical trial registration	None.
Study protocol	The full protocol can be accessed through the icddr,b ethical review committee.
Data collection	Samples were collected in Dhaka, Bangaldesh between Jan 1, 2017 and Dec 30, 2017
Outcomes	Participant blood samples at the time of vaccination (baseline) and at 90 days after vaccination were collected for enzyme-linked immunospot (ELISPOT) assay of o-specific polysaccharide memory B cells. For this study, samples were assessed as memory B cell (MBC) responder or non-responder by the increase or lack of change, respectively, in ELISPOT response. This metadata is provided in the manuscript as Supplementary Data.

Plants

Seed stocks	*Report on the source of all seed stocks or other plant material used. If applicable, state the seed stock centre and catalogue number. If plant specimens were collected from the field, describe the collection location, date and sampling procedures.*
Novel plant genotypes	*Describe the methods by which all novel plant genotypes were produced. This includes those generated by transgenic approaches, gene editing, chemical/radiation-based mutagenesis and hybridization. For transgenic lines, describe the transformation method, the number of independent lines analyzed and the generation upon which experiments were performed. For gene-edited lines, describe the editor used, the endogenous sequence targeted for editing, the targeting guide RNA sequence (if applicable) and how the editor was applied.*
Authentication	*Describe any authentication procedures for each seed stock used or novel genotype generated. Describe any experiments used to assess the effect of a mutation and, where applicable, how potential secondary effects (e.g. second site T-DNA insertions, mosiacism, off-target gene editing) were examined.*
